# NCAPG2 promotes prostate cancer malignancy and stemness via STAT3/c-MYC signaling

**DOI:** 10.1186/s12967-023-04834-9

**Published:** 2024-01-02

**Authors:** Enchong Zhang, Zhengjie Chen, Wangmin Liu, Lin Lin, Lina Wu, Johnny Guan, Jianfeng Wang, Chuize Kong, Jianbin Bi, Mo Zhang

**Affiliations:** 1 Department of Urology, Shenjing Hospital of China Medical University, Shenyang, China; 2 Department of Urology, The First Hospital of China Medical University, Shenyang, China; 3 Institute of Urology, China Medical University, Shenyang, China; 4 Department of Urology, University of California, Los Angeles, Los Angeles, CA USA; 5 Department of Laboratory Medicine, Shengjing Hospital of China Medical University, Shenyang, China

**Keywords:** Prostate cancer, NCAPG2, Cancer stemness, STAT3/c-MYC axis, Prognostic markers

## Abstract

**Background:**

Prostate cancer (PCa) is the second leading cause of cancer-related mortality among men worldwide, and its incidence has risen substantially in recent years. Therefore, there is an urgent need to identify novel biomarkers and precise therapeutic targets for managing PCa progression and recurrence.

**Methods:**

We investigated the clinical significance of NCAPG2 in PCa by exploring public datasets and our tissue microarray. Receiver operating characteristic (ROC) curve and survival analyses were performed to evaluate the correlation between NCAPG2 and PCa progression. Cell proliferation, wound healing, transwell, flow cytometry, cell cycle, tumor sphere formation, immunofluorescence (IF), co-immunoprecipitation (co-IP), and chromatin immunoprecipitation (ChIP) assays were conducted to further elucidate the molecular mechanism of NCAPG2 in PCa. Subcutaneous and orthotopic xenograft models were applied to investigate the effects of NCAPG2 on PCa proliferation in vivo. Tandem mass tag (TMT) quantitative proteomics was utilized to detect proteomic changes under NCAPG2 overexpression.

**Results:**

NCAPG2 was significantly upregulated in PCa, and its overexpression was associated with PCa progression and unfavorable prognosis. Knockdown of NCAPG2 inhibited the malignant behavior of PCa cells, whereas its overexpression promoted PCa aggressiveness. NCAPG2 depletion attenuated the development and growth of PCa in vivo. TMT quantitative proteomics analyses indicated that c-MYC activity was strongly correlated with NCAPG2 expression. The malignancy-promoting effect of NCAPG2 in PCa was mediated via c-MYC. NCAPG2 could directly bind to STAT3 and induce STAT3 occupancy on the MYC promoter, thus to transcriptionally activate c-MYC expression. Finally, we identified that NCAPG2 was positively correlated with cancer stem cell (CSC) markers and enhanced self-renewal capacity of PCa cells.

**Conclusions:**

NCAPG2 is highly expressed in PCa, and its level is significantly associated with PCa prognosis. NCAPG2 promotes PCa malignancy and drives cancer stemness via the STAT3/c-MYC signaling axis, highlighting its potential as a therapeutic target for PCa.

**Supplementary Information:**

The online version contains supplementary material available at 10.1186/s12967-023-04834-9.

## Background

Prostate cancer (PCa) is among the most prevalent male malignancies worldwide [1–4]. Over 1.2 million new cases of PCa are diagnosed, and approximately 350,000 PCa-related deaths are reported each year globally, ranking PCa among the leading causes of cancer-related death in men [1, 5, 6]. While indolent diseases have slowly progressing tumors with little or no clinical manifestation, patients with aggressive PCa may suffer from an invariably lethal outcome [7]. Currently employed treatment approaches for PCa include surgery, radiotherapy, ablation, androgen castration, chemotherapy, and immunotherapy [7]. Despite the rapid development of therapeutic strategies, most men succumb to tumor recurrence and distant metastases [8]. The mechanisms of PCa progression are highly complex and involve multiple molecular processes. Therefore, identifying novel druggable targets to facilitate the development of effective therapies are urgently needed.

Non-SMC condensin II complex subunit G2 (NCAPG2), which is a subunit of the chromosome condensin II complex, plays a critical role in chromosome condensation and segregation during mitosis [9, 10]. It has been demonstrated that NCAPG2 could mediate polo-like kinase 1 (PLK1) localization to the kinetochore during the process of microtubule attachment to facilitate chromosome segregation [10]. Accumulating evidences suggest that aberrant NCAPG2 expression contributes to oncogenesis in several human malignancies [11–13]. NCAPG2 was found to facilitate the proliferation, migration, and invasion of glioblastoma cells, in addition to regulating the G1/S phase [11]. Currently, there is a growing consensus that cell cycle dysregulation is crucial for both PCa initiation and progression [14, 15]. Notably, another non-SMC regulatory subunit of Condensin II complex, NCAPD3, has been recently reported to promote PCa occurrence and progression [16, 17]. However, the exact role and molecular mechanisms of NCAPG2 in PCa remain unexplored.

Cancer stem cells (CSCs) have been shown to participate in the PCa progression and metastasis [18, 19]. Nevertheless, the origin and characterization of CSC state in PCa still keeps controversial [20]. The MYC family of proto-oncogenes encodes a group of multi-functional transcription factors whose deregulation play an essential role in the initiation and maintenance of various cancers [21]. MYC is commonly overexpressed and amplified in PCa [22]. In particular, recent study has implicated MYC as a critical oncoprotein required for the stemness and tumorigenesis in breast cancer [23]. However, there is no known association for NCAPG2 with MYC, stemness in PCa progression.

In the present study, we aimed to elucidate the role and underlying mechanism of NCAPG2 in PCa. First, association of NCAPG2 with PCa progression and prognosis was assessed. Next, we found that NCAPG2 promoted PCa malignant biological properties via up-regulation of c-MYC. NCAPG2 has been demonstrated to bind with STAT3 and induce STAT3 occupancy on the MYC promoter, thus to subsequently activate c-MYC expression. Finally, given the link between c-MYC and cancer stemness, we identified that NCAPG2 was positively correlated with PCa CSC properties.

## Methods

### Patient tissue specimens

In this study, RNA-seq data of 497 PCa and 52 para-carcinoma tissues from The Cancer Genome Atlas (TCGA) (https://portal.gdc.cancer.gov/) as well as of 100 normal prostate tissues from The Genotype-Tissue Expression (GTEx) (https://gtexportal.org/home/) were downloaded. All RNA-seq data were transformed as transcripts per million (TPM), and log2 transformation was performed. A tissue microarray (Cat No.: HProA150PG02, Lot No.: XT19-024) containing 150 tissues (three normal prostate tissues, 52 para-carcinoma tissues, and 95 PCa tissues) was purchased from Shanghai Outdo Biotech Company. Furthermore, five pairs of PCa tissues and corresponding adjacent tissues were obtained from the Shengjing Hospital of China Medical University (clinical information is shown in Additional file 2: Table S1). GSE70769 and GSE116918 were obtained using the GEOquery R package from the Gene Expression Omnibus (GEO) database [24, 25]. The DKFZ2018 and MSKCC2010 datasets were downloaded from the cBioPortal database [26–28]. Patients with incomplete survival data or a follow-up duration of less than 30 days were excluded. The detailed clinical information of the above public datasets has been displayed in Additional file 2: Table S2.

### Cell culture and transfection

Three PCa cell lines (LNCaP, DU145, and PC3) and one prostate stromal cell line (WPMY-1) were purchased from the National Collection of Authenticated Cell Cultures in Shanghai, China. Additionally, NCI-H660 PCa cell lines were generously provided by Dr. Robert E. Reiter from the University of California, Los Angeles. LNCaP and DU145 cells were cultured in RPMI-1640 medium containing 10% fetal bovine serum (FBS), while PC-3 cells were cultured in the Dulbecco modified Eagle medium (DMEM) with 5% FBS. WPMY-1 cells were maintained in DMEM medium containing 10% FBS. NCI-H660 cells were cultured in RPMI1640 medium with 5% FBS, 10 nmol/L β-estradiol, 10 nmol/L hydrocortisone, 1% insulin-transferrin-selenium, and 2 mmol/L l-glutamine. All cells were maintained in a humidified incubator at 37 ℃ with 5% CO_2_. We acquired NCAPG2 shRNA, NCAPG2 plasmids, c-MYC plasmids, and lentivirus from Shanghai Biosciences Co., Ltd. (Biosciences, Shanghai, China), and their specific sequences are detailed in Additional file 2: Table S3. Transfection was carried out according to the manufacturer’s protocol. We employed puromycin (Gibco, USA) at a concentration of 1 μg/mL to select stably transfected cell lines. The c-MYC inhibitor 10,058-F4 and STAT3 inhibitor C188-9 were procured from Selleck USA and utilized in accordance with the manufacturer’s instructions.

### Receiver operating characteristic (ROC) curve

ROC curve analysis was performed using the pROC R package and visualized using the ggplot2 R package to evaluate the diagnostic value of NCAPG2. The prognostic accuracy of NCAPG2 was evaluated using a time-dependent ROC (tdROC) curve. The tdROC analysis was conducted using the survivalROC R package.

### Survival analysis

The log-rank test and Cox regression were used to calculate *P* values using the survival R package. Survival curves were plotted using the survminer R package. The outcome events in survival analysis were progression-free interval (PFI) and biochemical recurrence (BCR), all based on the clinical information in the different datasets. PFI referred to the period from the date of diagnosis until the date of the first occurrence of a new PCa-related event, which includes progression of the disease, locoregional recurrence, distant metastasis, or death with tumor [29]. BCR was defined as two sequential PSA values ≥ 0.2 ng/mL after radical prostatectomy or radiotherapy [30].

### Western blot (WB) assay

Sample protein concentration was determined using a BCA protein assay kit (Beyotime, Shanghai, China) following the manufacturer's instructions. The primary antibodies used in our study are listed in Additional file 2: Table S4, along with their dilutions and sources. HRP-conjugated AffiniPure goat anti-mouse and anti-rabbit IgG antibodies were purchased from Proteintech (Wuhan, China) and used at appropriate dilutions. Protein bands were visualized using a High-sig ECL Western Blotting Substrate (Tanon, Shanghai, China) and imaged using an imaging system (Bio-Rad, Hercules, CA, USA).

### Immunohistochemistry (IHC)

Fresh tissues were fixed in 4% paraformaldehyde (Solarbio, Beijing, China) for over 24 h. The fixed tissues were dehydrated by placing them in phosphate-buffered saline (PBS), gently washed for 1 min, packed and marked in embedding boxes, and then passed through the tank of different ethanol concentrations. Tissues were cleared by immersion into tanks containing xylene I and II for 20 min each. Subsequently, specimens were sectioned, counterstained with hematoxylin and eosin (H&E), and mounted. Four-mm-thick tissue sections were also used for IHC staining with an IHC kit (Sangon Biotech, Shanghai, China). The detailed information of primary antibodies are provided in Additional file 2: Table S4. To quantify the staining intensity, ImageJ software was applied to capture the IHC images using a microscope (Olympus, Tokyo, Japan) at 20X magnification. Next, the images were converted to grayscale and the staining intensity was measured using the integrated density method. Briefly, the region of interest (ROI) was selected, and average gray value of the ROI was then subtracted from the background. The integrated density was calculated by multiplying the area of the ROI by the mean gray value. To ensure consistency, all images were processed using the same threshold settings. The median staining intensity of NCAPG2 was used as the cutoff value to classify the samples into the low-expression and high-expression group.

### Quantitative real-time polymerase chain reaction (qPCR)

Total RNA was extracted using TRIzol reagent (Merck, Germany). The HiScript^®^ II Q RT SuperMix for qPCR (+gDNA wiper) was used for reverse transcription (Vazyme, Nanjing, China). The AceQ qPCR SYBR Green Master Mix (Vazyme, Nanjing, China) was used for qPCR. Two-step qPCR was performed with the following program settings: hold mode, 95 ℃ for 60 s, once; two-step PCR mode, 95 ℃ for 10 s, 60 ℃ for 30 s, 45 cycles; dissociation mode, 95 ℃ 15 s, 55 ℃ for 60 s, 95 ℃ for 15 s, once. The Ct values were then calculated, and the relative mRNA expression of target genes was determined using the 2^−ΔΔCt^ method. Primer sequences are listed in Additional file 2: Table S5.

### Cell proliferation assays

Cells were seeded in a 96-well plate at a density of 2000 cells/well (100 μL/well) and counted using Celigo once a day for five consecutive days. Fluorescence microscopy was employed to scrutinize the expression of green fluorescent protein (GFP) within cells. For Cell Counting Kit-8 (CCK-8) assay (Dojindo, Japan), cells were seeded in 96-well plates at a density of 5000 cells/well, with three replicates for each group of samples. The edges of 96-well plates were then sealed with sterile PBS. The absorbance value of samples at 450 nm was detected every 24 h, and the experiment lasted for 96 h. For the colony formation assay, cells were seeded in 6-well plates at a density of 500 cells/well. After 15 days of culture, the experiment was terminated, and the cells were stained with 0.1% crystal violet solution for 20 min.

### Wound healing and transwell invasion assays

For the wound healing assay, cells were seeded in a six-well plate, and a line was scratched when the confluence reached 90–100%. The cells were imaged under a microscope. Cell migration was observed under a microscope after 24 h, and images were captured at the location of the scratch.

For transwell invasion assays, serum-free medium was used to prepare the cell suspension, and the cell concentration was adjusted to 2 × 10^5 ^cells/mL. Matrigel (Corning, USA) was seeded into the transwell chamber, after which 100 μL cell suspension was added to the upper chamber. After 24 h of culture, the cells were stained with 0.1% crystal violet solution for 20 min. Five random visual fields were imaged using a microscope.

### Flow cytometry and cell cycle assays

For stem cell marker analysis, the PCa cells were harvested following transfection. Approximately 10^6^ cells were incubated with CD44 antibodies (0.5 µg) for 30 min at 4 ℃. CD44 staining was performed using a PE-conjugated anti-CD44 antibody (BD Pharmingen, San Diego, CA, USA). The cells were then resuspended in 500 µL of binding buffer and analyzed using a flow cytometer (Aceabio, USA). PI staining (Sigma, Germany) was utilized to analyze cell cycle distribution in different experimental groups. Prior to staining, the cell precipitates were washed twice with PBS. Additionally, a 300-µM mesh screen was employed to filter cells during the flow cytometry analysis.

### Mouse experiments

All animal experiments were approved by the Ethics Committee of the Shengjing Hospital of China Medical University. Five- to 8-week-old male athymic nude mice and C57BL/6 mice were purchased from Beijing HFK Bioscience Co. Ltd. DU145 cells initially collected via centrifugation were used to establish the subcutaneous tumor model. Cell suspensions were prepared with PBS, and the cell concentration was adjusted to 1 × 10^7^ cells/mL. A 0.2 mL cell suspension was injected subcutaneously into the right flank of skin in 12 nude mice. The body weight of nude mice and the length of the long and short axes of subcutaneous tumors were recorded every 5 days from the first day of injection. Tumor volume was measured using a digital caliper (V = length × width^2^/2). After 35 days, the mice were euthanized according to the protocol of the Animal Research Committee.

For the orthotopic tumor model, 5 × 10^4^ DU145 cells (sh-NCAPG2 and sh-NC) in 5 μL PBS/Matrigel (1:1, v/v, Corning, USA) were injected into the dorsal prostate lobes in C57BL/6 mice (n = 6). Tumor growth was monitored using bioluminescence imaging (BLI). BLI was performed with the IVIS Lumina II In Vivo Imaging System (PerkinElmer, MA, USA), as described in our previous work [31].

### Tandem mass tag (TMT) quantitative proteomics

LNCaP cells overexpressing NCAPG2 or transfected with an empty vector were sampled for three biological replicates. The amount of protein was quantified using the BCA Protein Assay Kit (Bio-Rad, USA). The protein was then digested according to the filter-aided sample preparation (FASP) procedure described by Mann et al. [32]. The peptide mixture of each sample was labeled with TMT reagent according to the manufacturer’s guidelines (Thermo Fisher Scientific). Finally, liquid chromatography-tandem mass spectrometry (LC–MS/MS) was used to obtain raw data for each sample. The MS raw data for each sample were searched using the MASCOT engine (Matrix Science, London, UK; version 2.2) embedded in Proteome Discoverer 1.4 software for identification and quantitation analysis. To conduct hierarchical clustering analysis, Cluster 3.0 (http://bonsai.hgc.jp/~mdehoon/software/cluster/software.htm) and Java Treeview software (http://jtreeview.sourceforge.net) were used. Finally, enrichment analysis was conducted based on the Kyoto Encyclopedia of Genes and Genomes (KEGG) database (http://geneontology.org/).

### Gene set enrichment analysis (GSEA)

GSEA was used to search for gene sets associated with NCAPG2 expression. Background gene sets were used as “Hallmark Gene Sets” in the Molecular Signatures Database [33, 34] (MSigDB) V7.2 (https://www.gsea-msigdb.org/gsea/msigdb/). Spearman correlation analysis was conducted to calculate the correlation between the expression levels of other genes and *NCAPG2* in PCa patient data from the TCGA database. Each gene was arranged in descending order based on the calculated correlation coefficient. The ClusterProfiler R package was utilized to perform GSEA analysis. Settings during operation were as follows: exponent = 1, nPerm = 1000, minGSSize = 10, maxGSSize = 500, pvalueCutoff = 0.25, pAdjustMethod = "BH,” verbose = TRUE, seed = TRUE, by = “fgsea.”

### Sphere formation assay

As described previously, the PCa cell suspension was mixed with Matrigel (Corning, USA) and transferred to ultra-low attachment 96-well plates (Corning, USA) at a density of 200 cells/well for suspension culture to form spheres [35]. The serum-free culture medium consisted of DMEM:Ham’s F12 (1:1) (BL305A, Biosharp, China) supplemented with 20 ng/mL epidermal growth factor (EGF, 10,605-HNAE, Sino Biological Inc., China), 10 ng/mL basic fibroblast growth factor (bFGF, 10014-HNAE, Sino Biological Inc., China), and 2% B27 supplement (17504044, Thermo Fisher Scientific, USA). Spheres were evaluated for the number and size by light microscopy 14 days after incubation. The enumeration of tumor spheres (diameter > 50 µm) was performed subsequently.

### Immunofluorescence (IF) assay

LNCaP cells were pre-treated with IL-6 (R&D Systems, #206-IL-050) at a concentration of 20 ng/mL for 24 h. Then LNCaP and DU145 cells were fixed with 4% paraformaldehyde for 10 min, permeabilized with 0.1% Triton X-100 for 15 min, and blocked with 2% BSA for 1 h at room temperature. Both cells were incubated with primary antibodies against NCAPG2 and c-MYC overnight at 4 ℃. CoraLite488-conjugated Goat Anti-Rabbit IgG (H+L) (Proteintech, China) and CoraLite594– conjugated Goat Anti-Mouse IgG (H+L) (Proteintech, China) secondary antibodies were used at a dilution of 1:500 and incubated 1 h at room temperature. DAPI staining solution (Beyotime, China) was used to counterstain nuclei. Images were captured using a confocal microscope (Olympus, FV3000).

### Co-immunoprecipitation (co-IP)

Cells were washed with chilled PBS. Subsequently, total protein was extracted using the Pierce^™^ Classic Magnetic IP/Co-IP Kit (Thermo Scientific, USA), according to the manufacturer’s instructions, and subjected to co-IP with anti-NCAPG2 and anti-STAT3 primary antibodies. Detailed information on the primary antibodies is provided in Additional file 2: Table S4. The co-IP experiment was also performed using the Pierce^™^ Classic Magnetic IP/Co-IP Kit.

### Chromatin immunoprecipitation (ChIP)

Soluble chromatin was precipitated using primary antibodies (Additional file 2: Table S4). The precipitates were analyzed by qPCR using the AceQ qPCR SYBR Green Master Mix (Vazyme, Nanjing, China) and an ABI Prism 7300 sequence detector (Applied Biosystems). Data are displayed as relative fold-change to IgG. Primers used for ChIP qPCR are listed in Additional file 2: Table S6.

### Statistical analysis

All experiments were performed in triplicate, and error bars representing the mean plus standard deviation were included in all statistical graphs. Data analysis and visualization were performed using R software (version 3.6.3), with the relevant packages identified in the corresponding section. As gene sequencing data were not normally distributed, the Wilcoxon test was used to determine statistical differences in continuous variables between the two groups. Differences between the two groups were evaluated using the Student’s t-test. The sign test or Mann–Whitney *U*-test were performed to analyze differences in discrete variables between or among groups. *P* < 0.05 was defined as the threshold of statistical significance.

## Results

### NCAPG2 is highly expressed in PCa tissues and has significant clinical relevance

We first analyzed NCAPG2 expression using PCa samples from the TCGA database. The expression of NCAPG2 was higher in 497 PCa tissues compared to that in 52 normal tissues (Wilcoxon test *P* < 0.001, Additional file 1: Figure S1a). To eliminate the variance between samples, we subsequently compared NCAPG2 expression in 52 pairs of PCa and corresponding para-cancerous tissues. As shown in Additional file 1: Figure S1b, NCAPG2 expression was significantly higher in PCa tissues (Wilcoxon test, *P* < 0.001). To further validate our findings in a larger sample, normal prostate samples data from the GTEx database were combined with normal samples from TCGA PCa cohort. In accordance with previous results, NCAPG2 was shown to be still highly expressed in PCa tissues (Wilcoxon test, *P* < 0.001, Additional file 1: Figure S1c).

Next, the correlation between NCAPG2 and clinicopathological data of PCa patients were explored according to TCGA database (Additional file 2: Table S7). NCAPG2 expression increased with the T stage, N stage, Gleason score, and serum PSA level (Wilcoxon test *P* < 0.01, Additional file 1: Figure S1 d-g). In addition, we found that NCAPG2 expression was upregulated in PCa patients with poor response after primary treatment (CR: complete response; PR: partial response; SD: stable disease; PD: progressive disease) (Wilcoxon test *P* < 0.001, Additional file 1: Figure S1h). Patients with residual tumors post-surgery also presented with NCAPG2 overexpression (R0: no residual tumor; R1: microscopic residual tumor; R2: macroscopic residual tumor) (Additional file 1: Figure S1i; Wilcoxon test, *P* < 0.05). Meanwhile, NCAPG2 expression was higher in patients with worse disease-specific survival (DSS) and PFI (Wilcoxon test *P* < 0.001, Additional file 1: Figure S1j-k). Moreover, the diagnostic value of NCAPG2 to PCa was examined. ROC analysis performed using TCGA and GTEx data showed an area under curve (AUC) of 0.706 (Additional file 1: Figure S2a), while using TCGA PCa data alone provided an AUC of 0.659 (Additional file 1: Figure S2b).

**Fig. 1:**
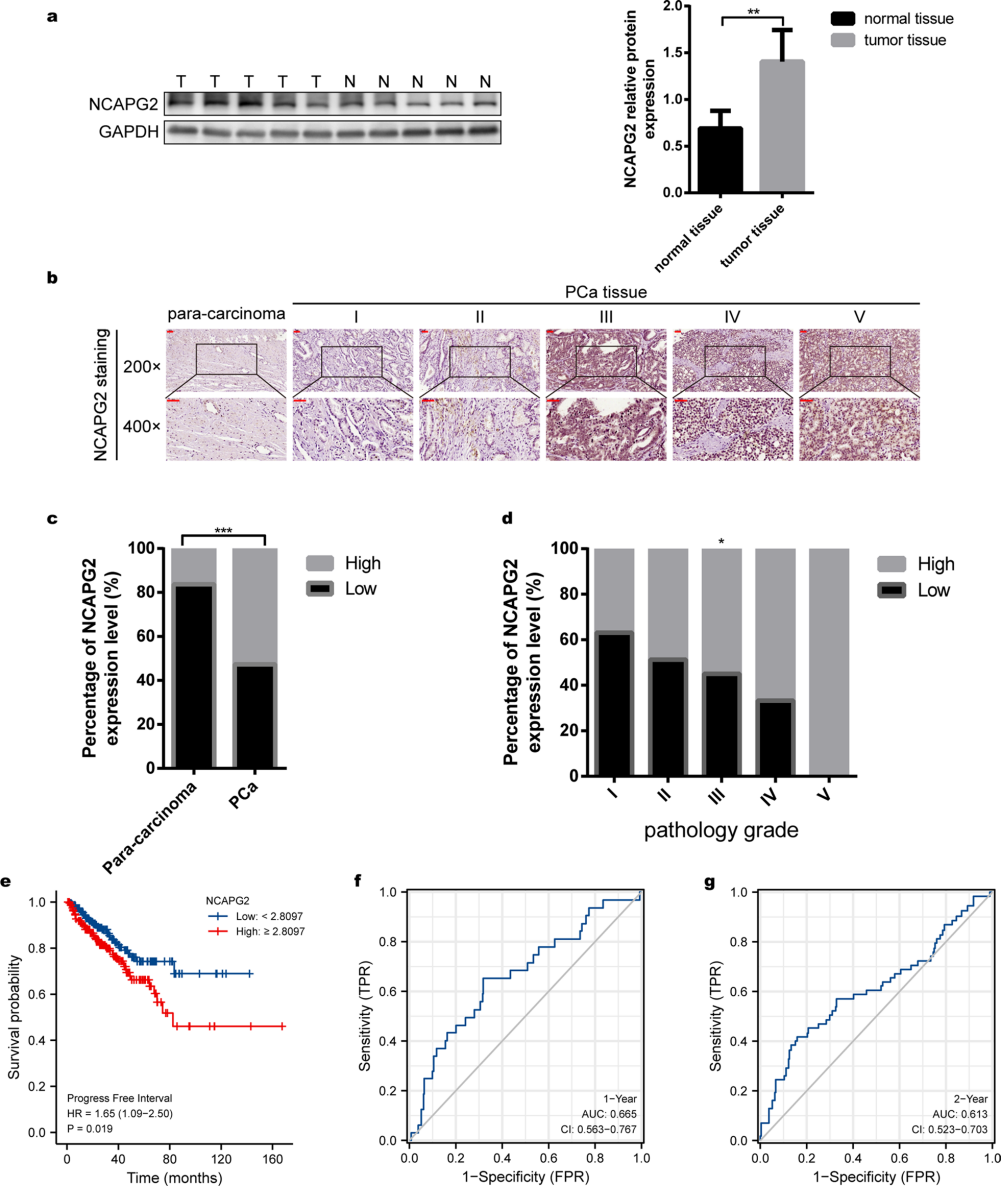
NCAPG2 is highly expressed in PCa tissues and is a promising prognostic biomarker for PCa. **a** WB analysis validated that NCAPG2 protein was overexpressed in prostate tumors (T) compared to normal (N) benign prostate tissues. **b** Representative images of NCAPG2 IHC staining in para-carcinoma tissues and PCa tissues with different ISUP grades. **c** Statistical analysis of IHC assay showed that NCAPG2 expression in PCa tissues was significantly higher than that in adjacent tissues. **d** NCAPG2 expression increased with the pathological ISUP grades. **e** PFI survival analysis of the PCa patients with high- or low- NCAPG2 expression in TCGA database. **f**, **g** The tdROC indicated that the level of NCAPG2 could effectively predict the 1-year and 2-year PFI survival, respectively. *PCa* prostate cancer, *WB* western blot, *IHC* immunohistochemical staining, *ISUP* international Society of Urological Pathology, *tdROC* time-dependent receiver operating characteristic curve. * means *P* < 0.05, ** means *P* < 0.01, *** means *P* < 0.001, ns means *P* > 0.05, and P < 0.05 is defined as statistically significant

In our cohort samples, WB analysis validated that NCAPG2 expression was higher in PCa tissues than in normal prostate tissues (Student’s t-test *P* < 0.01; Fig. 1a). Subsequently, the tissue microarray was subjected to IHC assays. Independent evaluation by two experienced pathologists revealed that the level of NCAPG2 in PCa tissues (n = 93) was significantly higher than that in adjacent normal tissues (n = 43) (sign test *P* < 0.001, Fig. 1b, c). There was a significant difference in NCAPG2 level based on the pathological grading proposed by the International Society of Urological Pathology (Mann–Whitney *U* analysis *P* < 0.05, Fig. 1d). Representative images of hematoxylin–eosin staining of PCa and para-carcinoma tissue sections were provided in Additional file 1: Figure S3.

Finally, the prognostic value of NCAPG2 was evaluated. In the TCGA database, the median value of 2.8097 for the expression level of NCAPG2 was defined as the cutoff value to classify the PCa patients into a low-expression group and a high-expression group. High NCAPG2 expression was associated with a poor PFI (Cox *P* < 0.05, Fig. 1e). The tdROC analysis indicated that NCAPG2 expression could effectively predict the 1- (AUC = 0.665, Fig. 1f), 2- (AUC = 0.613, Fig. 1g), 3- (AUC = 0.605, Additional file 1: Figure S2c), 6- (AUC = 0.665, Additional file 1: Figure S2d), 8- (AUC = 0.666, Additional file 1: Figure S2e), and 10-year PFI (AUC = 0.713, Additional file 1: Figure S2f). Furthermore, patients with high NCAPG2 expression exhibited poor BCR survival in DKFZ2018 (log-rank test *P* < 0.001, Additional file 1: Figure S2g), GSE70769 (log-rank test *P* = 0.009, Additional file 1: Figure S2h) and MSKCC2010 dataset (log-rank test *P* < 0.001, Additional file 1: Figure S2i).

Collectively, our analyses demonstrated that NCAPG2 was upregulated in PCa tissues, and its levels were significantly associated with PCa progression. Furthermore, NCAPG2 expression had the potential to predict PCa prognosis.

### Knockdown of NCAPG2 inhibits the malignant behavior of PCa cells in vitro

**Fig. 2:**
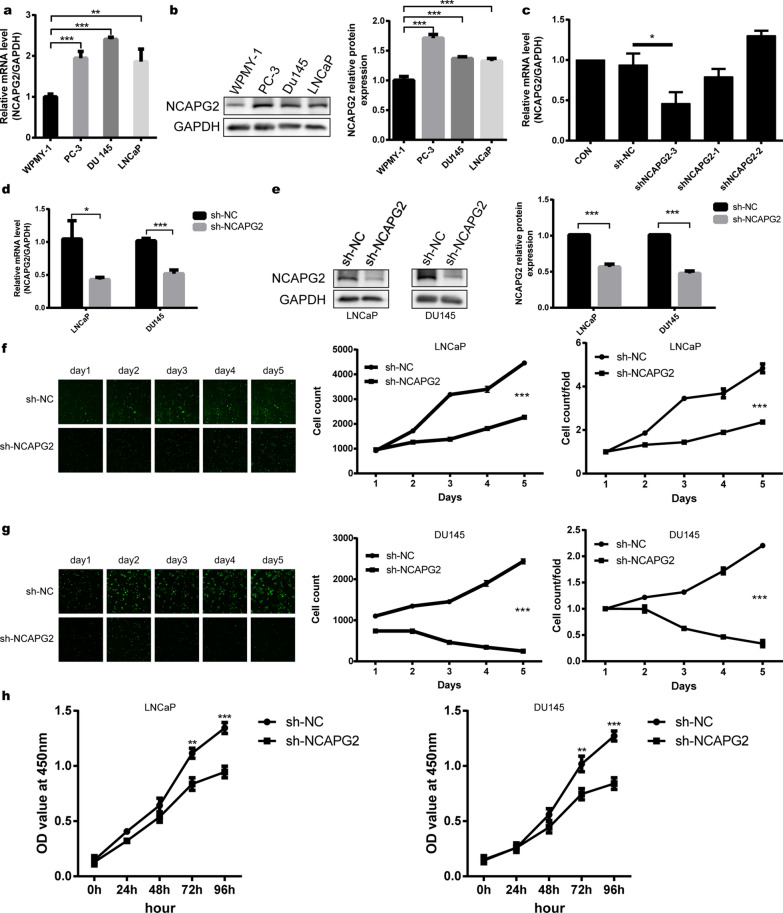
NCAPG2 is highly expressed in PCa cells and knockdown of NCAPG2 inhibits PCa cell proliferation in vitro. **a**, **b** Compared with WPMY-1 cells, levels of NCAPG2 mRNA (qPCR) and protein (WB) were highly expressed in PC-3, DU145 and LNCaP cells. **c** In DU145 cells, compared with the sh-NC group, the knockdown efficiency of NCAPG2 in shNCAPG2-3 group was 50.9%, whereas the shNCAPG2-1 group or shNCAPG2-2 group showed no significant change. **d**, **e** qPCR and WB analysis of NCAPG2 expression in LNCaP and DU145 cells after transfection of sh-NC or sh-NCAPG2. **f**–**h** LNCaP and DU145 cells proliferation were analyzed by Celigo cell counting and CCK-8 assay, following NCAPG2 knockdown. *PCa* prostate cancer, *qPCR* quantitative real-time polymerase chain reaction, *WB* western blot, *CCK-8* cell counting kit-8. *P* values were calculated by the Student’s t-test. * means *P* < 0.05, ** means *P* < 0.01, *** means *P* < 0.001, ns means *P* > 0.05, and *P* < 0.05 is defined as statistically significant

Compared with WPMY-1, the mRNA and protein levels of NCAPG2 were upregulated in PC-3, DU145, and LNCaP cells (Student’s t-test *P* < 0.01; Fig. 2a-b). qPCR analysis revealed no significant difference in NCAPG2 expression following lentiviral transduction in the shNCAPG2-1 and shNCAPG2-2 groups, whereas knockdown efficiency of NCAPG2 in the shNCAPG2-3 group was 50.9% (Student’s t-test *P* < 0.05, Fig. 2c). Therefore, shNCAPG2-3 was selected for subsequent experiments. As shown in Fig. 2d, knockdown efficiency of NCAPG2 mRNA in the sh-NCAPG2 group was 60.97% and 49.98% in LNCaP and DU145 cells (Student’s t-test *P* < 0.001), while a similar trend was observed in NCAPG2 protein levels (Student’s t-test *P* < 0.001; Fig. 2e). In addition, Celigo cell counting assay indicated that NCAPG2 knockdown significantly inhibited the proliferation in both LNCaP and DU145 cells (Student’s t-test *P* < 0.001; Fig. 2f-g). Likewise, downregulation of NCAPG2 in LNCaP and DU145 cells exhibited a slower proliferation rate in CCK-8 assays (Student’s t-test *P* < 0.001; Fig. 2h).

**Fig. 3:**
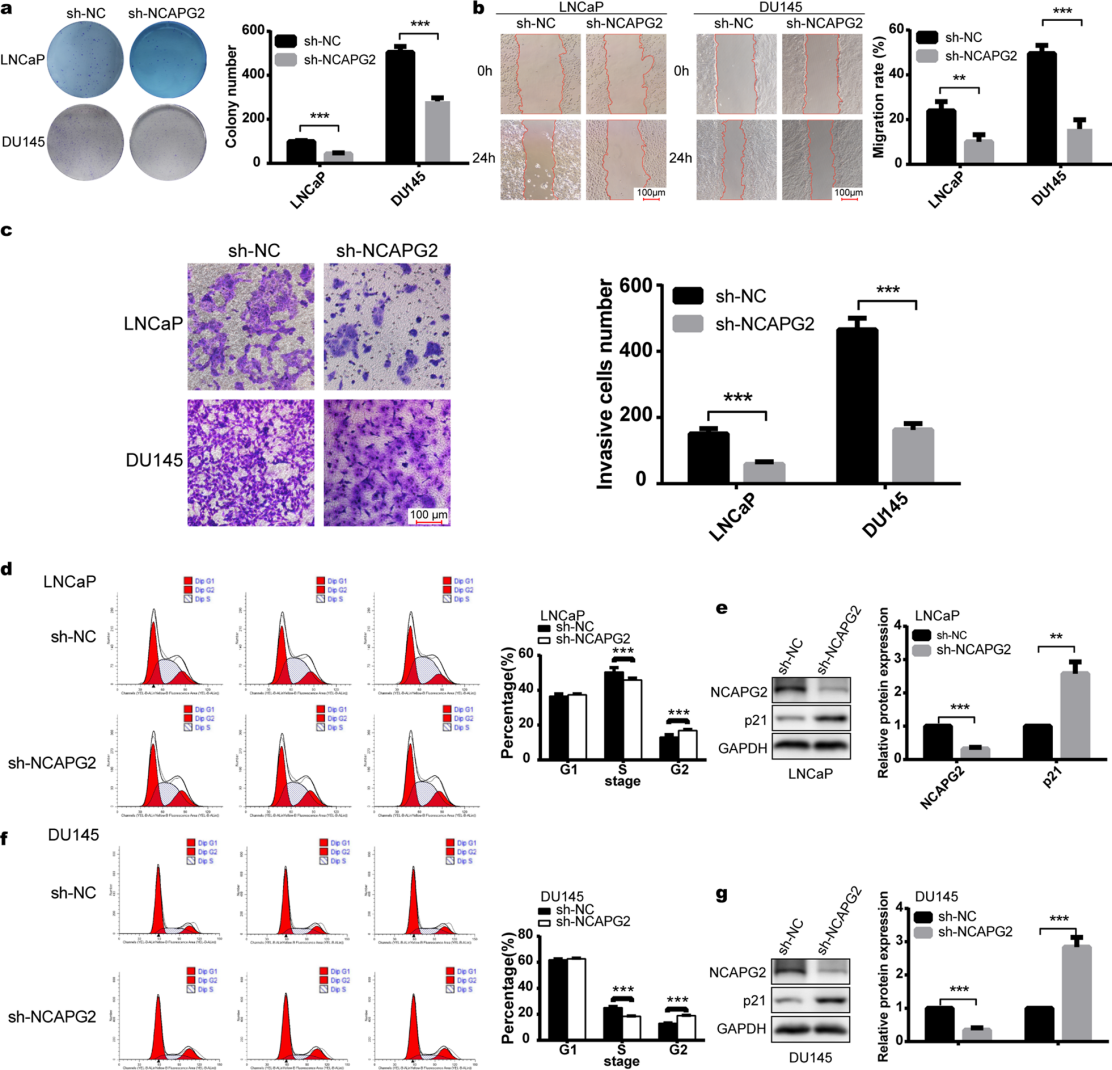
Knockdown of NCAPG2 suppresses PCa cell migration, invasion, and regulates cell cycle of PCa cells in vitro. **a** Colony formation assay showed that LNCaP and DU145 cells in sh-NCAPG2 group formed fewer colonies. **b**, **c** The migration rate and invasion ability of LNCaP and DU145 cells were determined by wound healing assay and transwell assay, respectively. **d–g** Cell cycle and WB analysis for LNCaP and DU145 cells transfected with sh-NC or sh-NCAPG2. Both two PCa cells showed an increase in the percentage of cells arrested in G2 phase with a concomitant decrease in S phase following NCAPG2 downregulation. WB assays indicated that knockdown of NCAPG2 led to a significant increase in the expression of p21 protein. *PCa* prostate cancer, *WB* western blot. *P* values were calculated by the Student’s t-test. * means *P* < 0.05, ** means *P* < 0.01, *** means *P* < 0.001, ns means *P* > 0.05, and *P* < 0.05 is defined as statistically significant

In the colony formation assay, LNCaP and DU145 cells in the sh-NCAPG2 group formed fewer colonies (Student’s t-test *P* < 0.001; Fig. 3a). As shown in Fig. 3b, the migration rate of cells were significantly decreased by 58% (Student’s t-test* P* < 0.01) and 68% (Student’s t-test* P* < 0.001) following NCAPG2 downregulation in LNCaP and DU145 cells, respectively. Transwell assays revealed that the invasion rate of sh-NCAPG2 group cells were reduced by 61% (Student’s t-test* P* < 0.001) and 65% (Student’s t-test* P* < 0.001) for LNCaP and DU145 cells, respectively (Fig. 3c). Furthermore, we found that NCAPG2 could regulate the cell cycle, showing an increase in the percentage of cells arrested in G2 phase with a concomitant decrease in S phase for NCAPG2-silencing LNCaP (Student’s t-test *P* < 0.001, Fig. 3d) and DU145 cells (Student’s t-test *P* < 0.05, Fig. 3f).

WB assays further demonstrated that knockdown of NCAPG2 led to a significant increase in the expression of cell cycle regulatory protein p21 (Fig. 3e and g).

### NCAPG2 overexpression promotes the malignant behavior of PCa cells in vitro

Next, we constructed NCAPG2 stable overexpression LNCaP and DU145 cell lines using plasmid-based approach. The overexpression efficiency of NCAPG2 were confirmed by qPCR and WB (Student’s t-test *P* < 0.001; Additional file 1: Figure S4a-b). CCK-8 assays revealed that LNCaP and DU145 cells with NCAPG2 overexpression exhibited a faster proliferation rate (Student’s t-test *P* < 0.01; Additional file 1: Figure S4c). In the colony formation assay, LNCaP (Student’s t-test *P* < 0.01, fold change = 1.5) and DU145 (Student’s t-test *P* < 0.001, fold change = 1.7) cells of the oe-NCAPG2 group formed more colonies (Additional file 1: Figure S4d). As shown in Additional file 1: Figure S4e, wound healing assay results indicated that the migration rate of PCa cells in the oe-NCAPG2 group (24 h) increased by 71% for LNCaP (Student’s t-test* P* < 0.05) and 165% for DU145 cells (Student’s t-test* P* < 0.001). In addition, the capabilities of invasion increased by 161% for LNCaP (Student’s t-test* P* < 0.001) and 39% for DU145 cells (Student’s t-test* P* < 0.05) after NCAPG2 overexpression (Additional file 1: Figure S4f).

### Targeting NCAPG2 inhibits PCa tumorigenicity in vivo

**Fig. 4:**
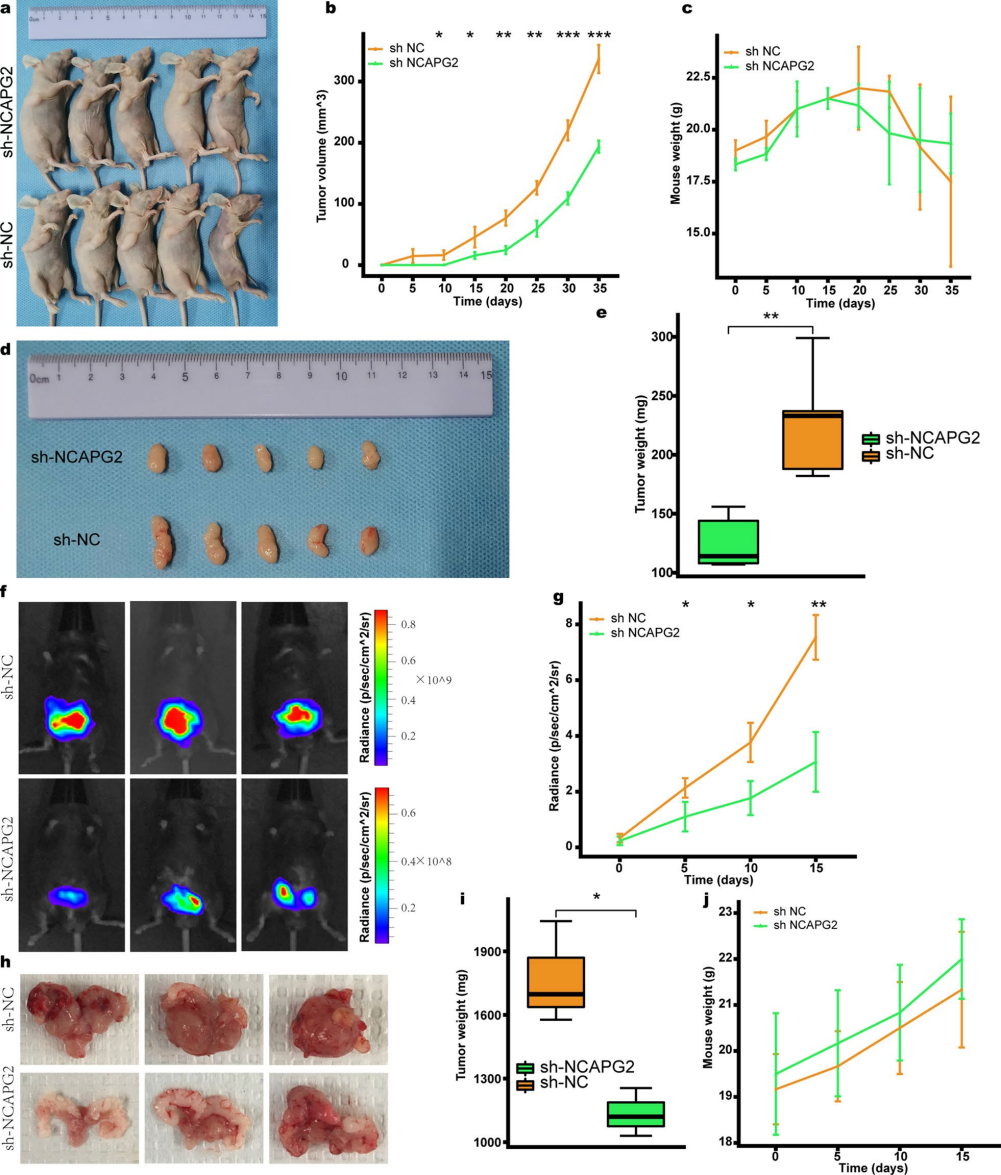
Targeting NCAPG2 inhibits the growth of PCa in vivo. **a** Representative images of subcutaneous xenograft derived from the sh-NCAPG2 and sh-NC DU145 cells. **b**, **c** Tumor volume and body weight in nude mice of sh-NCAPG2 and sh-NC group were measured every 5 days. **d**, **e** The size and weights of subcutaneous tumors in the sh-NCAPG2 group were significantly lower than the sh-NC group. **f** Representative bioluminescence images of orthotopic xenograft. **g** Tumor growth curves of orthotopic tumor in vivo were determined based on BLI radiance measured every 5 days. **h**, **i** The orthotopic tumors in sh-NCAPG2 group were smaller and lighter, compared with the sh-NC group. **j** Growth curve of body weight between the sh-NCAPG2 and sh-NC groups. *PCa* prostate cancer, *BLI* bioluminescence. *P* values were calculated by the Student’s t-test. * means *P* < 0.05, ** means *P* < 0.01, *** means *P* < 0.001, ns means *P* > 0.05, and *P* < 0.05 is defined as statistically significant

A subcutaneous xenograft model was initially established to investigate the role of NCAPG2 in PCa tumorigenesis in vivo. NCAPG2 downregulation strongly attenuated the growth rate of xenograft tumors in mice. As a result, the size and weights of the tumors were apparently lower in the sh-NCAPG2 group compared with that in the sh-NC group (Student’s t-test, *P* < 0.05), while there was no significant difference in mice body weight between the two groups (Fig. 4a-e). Subsequently, tumor samples were harvested from the mice. Histologic examination for tumors indicated that silencing NCAPG2 apparently impaired the tumor growth (Additional file 1: Figure S5a). WB analysis confirmed that NCAPG2 levels were significantly lower in the sh-NCAPG2 group than in the sh-NC group (Student’s t-test *P* < 0.001; Additional file 1: Figure S5b-c), while the expression of p21 protein were upregulated in the sh-NCAPG2 group compared with the sh-NC group (Student’s t-test *P* < 0.05; Additional file 1: Figure S5d-e). On the contrary, NCAPG2 overexpression significantly promoted the subcutaneous tumors growth, with no difference in mouse weight between the oe-NC and oe-NCAPG2 groups (Additional file 1: Figure S6a-e). WB results further demonstrated that NCAPG2 levels were significantly upregulated in the oe-NCAPG2 group (Student’s t-test, *P* < 0.01; Additional file 1: Figure S6f-g), while there was a remarkable reduction for p21 expression in the oe-NCAPG2 group (Student’s t-test, *P* < 0.05; Additional file 1: Figure S6h-i).

We then established an orthotopic xenograft model by injecting DU145 cells, with or without knockdown of NCAPG2, orthotopically into the dorsal prostate lobes of C57BL/6 mice. Tumor burden was measured using bioluminescence in vivo. Our results showed that tumor burden were significantly lower in the sh-NCAPG2 group compared with the sh-NC group (Student’s t-test, *P* < 0.05; Fig. 4f-g). After 15 days of tumor inoculation, the mice were euthanized and tumors were then dissected out. As shown in Fig. 4h, i, targeting NCAPG2 could significantly inhibit the size and weights of orthotopic tumor (Student’s t-test *P* < 0.05), while there was no significant difference in body weight between the experimental and control group (Fig. 4j).

### NCAPG2 positively regulates the c-MYC expression

**Fig. 5:**
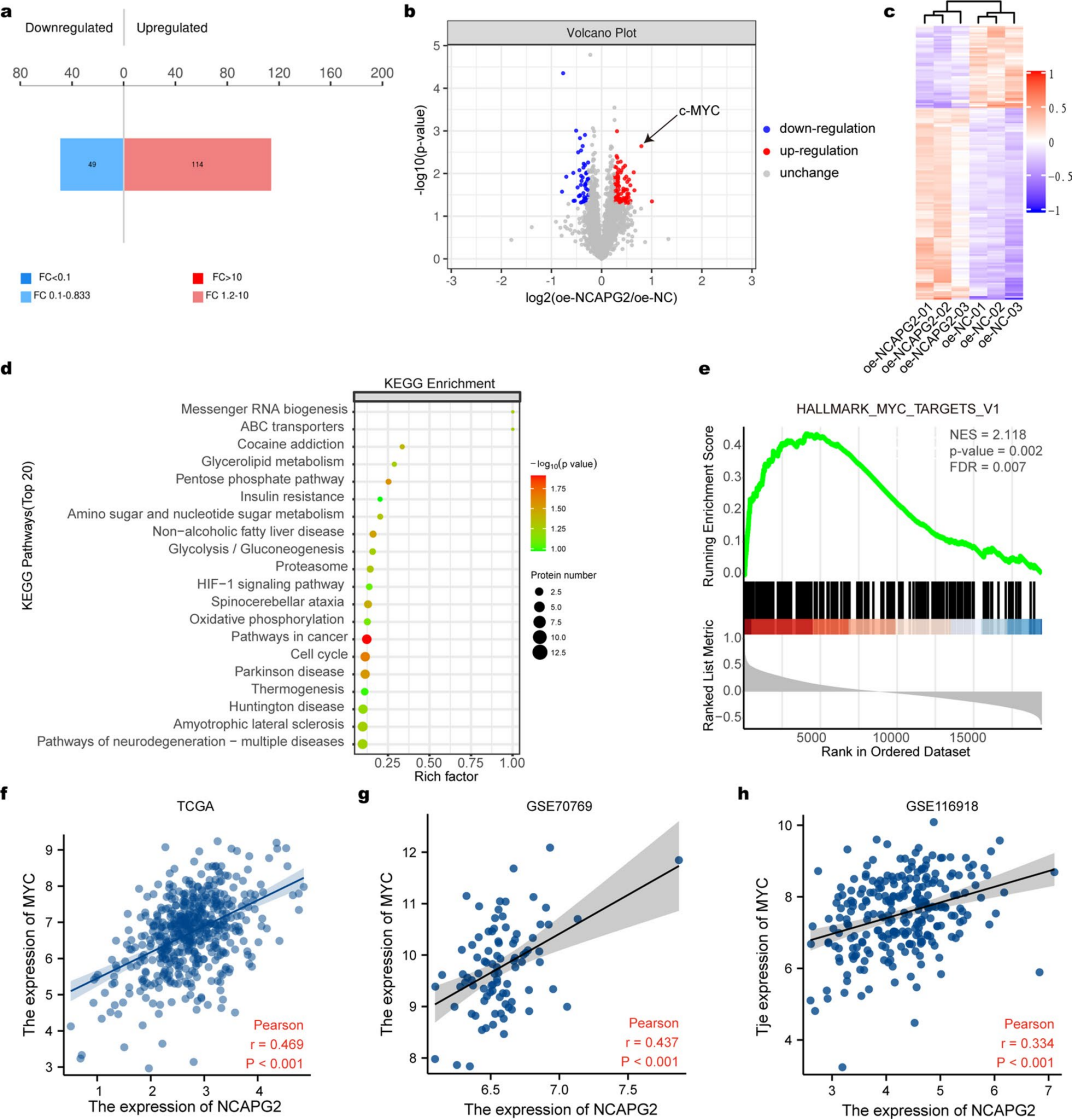
NCAPG2 is positively correlated with c-MYC expression. **a** The bar plot showed there were 114 up-regulated and 49 down-regulated proteins between oe-NCAPG2 and oe-NC groups. **b** LC-MS/MS analysis revealed that protein level of c-MYC was significantly increased in the oe-NCAPG2 group. **c** The hierarchical cluster indicated that there were significant differences in protein expression pattern between oe-NCAPG2 and oe-NC groups. **d** Top 20 terms of KEGG results indicated the related pathways about NCAPG2. **e** GSEA showed the activity of c-MYC was positively related to NCAPG2 expression. **f**-**h** The expression of NCAPG2 and MYC was positively correlated based on TCGA dataset, GSE70769 dataset, and GSE116918 dataset. *LC-MS* liquid chromatography–mass spectrometry, *KEGG* Kyoto Encyclopedia of Genes and Genomes, *GSEA* Gene Set Enrichment Analysis, *TCGA* The Cancer Genome Atlas. *P* values were calculated by the Student’s t-test, and *P* < 0.05 is defined as statistically significant

**Fig. 6:**
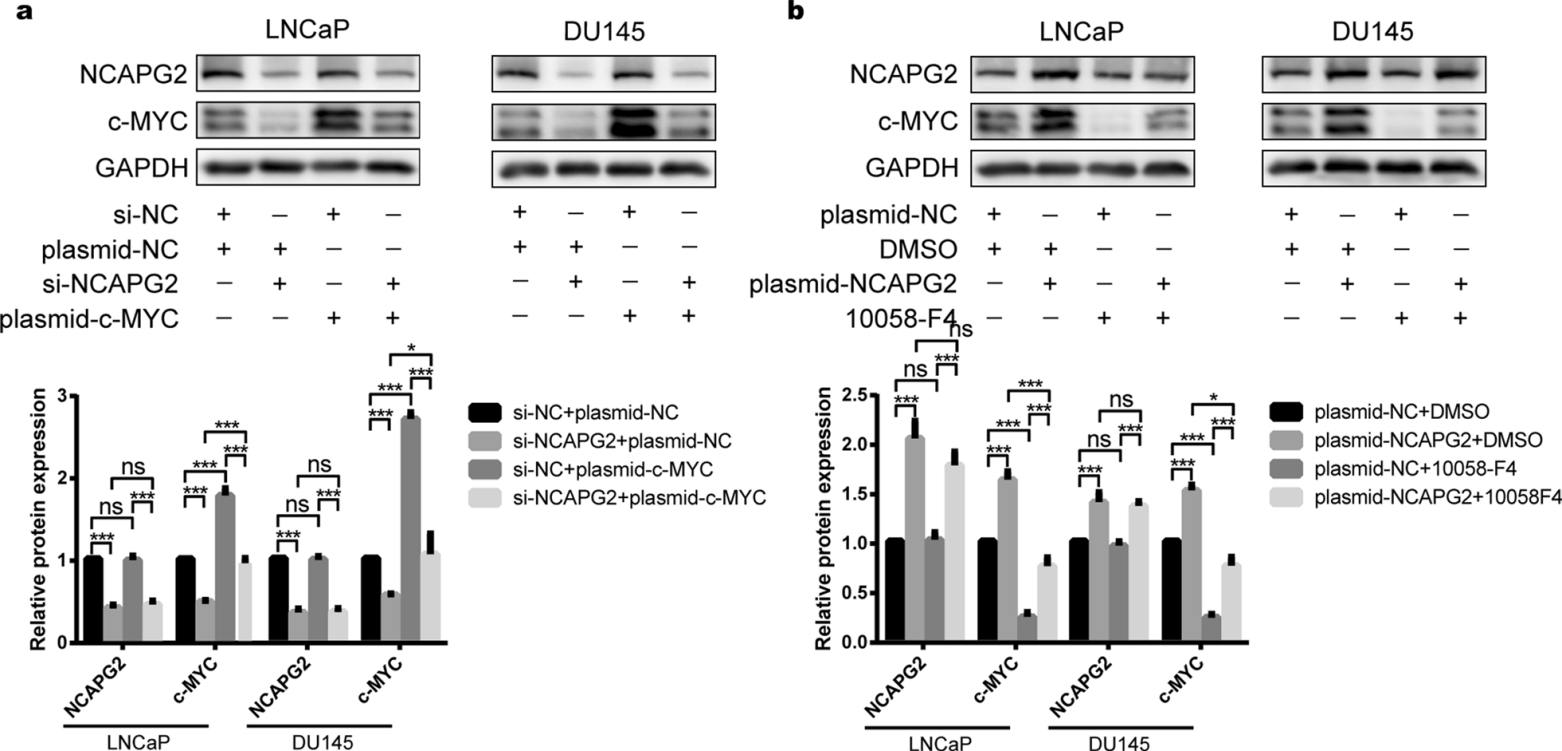
NCAPG2 could positively regulate the level of c-MYC. **a** WB showed that the expression of c-MYC was significantly suppressed following knockdown of NCAPG2, and NCAPG2 silencing could inhibit the effect of c-MYC overexpression plasmid in LNCaP and DU145 cells. **b** WB showed that the expression of c-MYC was significantly increased after NCAPG2 overexpression, and NCAPG2 upregulation could reverse the inhibitory effect of 10058-F4 in LNCaP and DU145 cells. *WB* western blot. *P* values were calculated by the Student’s t-test. * means *P* < 0.05, ** means *P* < 0.01, *** means *P* < 0.001, ns means *P* > 0.05, and *P* < 0.05 is defined as statistically significant

To elucidate the molecular mechanism underlying the role of NCAPG2 in PCa malignancy, TMT quantitative proteomics was performed. The results revealed 114 upregulated and 49 downregulated proteins in the oe-NCAPG2 group. Importantly, c-MYC represents one of the most significantly upregulated proteins with NCAPG2 overexpression (Student’s t-test *P* < 0.05, Fig. 5a-b, Additional file 2: Table S8). As shown in Fig. 5c, hierarchical cluster analysis indicated a significant difference between the oe-NCAPG2 and oe-NC groups. KEGG analysis of the upregulated proteins yielded “Pathways in cancer” and “Cell cycle” as enriched terms for c-MYC (Fig. 5d). Additionally, GSEA showed that c-MYC activity was positively correlated with NCAPG2 expression (Fig. 5e). In addition, we demonstrated that NCAPG2 and MYC expression were positively correlated in TCGA data (Pearson correlation test *P* < 0.001, rho = 0.469), GSE70769 (Pearson correlation test *P* < 0.001, rho = 0.437), and GSE116918 dataset (Pearson correlation test *P* < 0.001, rho = 0.334), as shown in Fig. 5f–h. WB results further confirmed that NCAPG2 could promote c-MYC levels. In LNCaP and DU145cells, NCAPG2 knockdown significantly reduced the levels of c-MYC, whereas NCAPG2 upregulation led to a significant increase in the c-MYC expression (Student’s t-test *P* < 0.001, Fig. 6). To perform rescue experiments, we set up experimental groups with c-MYC overexpression under NCAPG2 knockdown or 10058-F4 (c-MYC inhibitor) treatment under NCAPG2 overexpression, respectively. As expected, the results demonstrated that NCAPG2 silencing significantly inhibit the enhancement effect of c-MYC plasmid, while NCAPG2 overexpression could reverse the inhibitory effect of 10058-F4 in PCa cells (Student’s t-test, *P* < 0.05; Fig. 6a-b). Collectively, our findings support that NCAPG2 could positively regulate c-MYC levels.

### NCAPG2 promotes the malignant biological behavior of PCa via c-MYC

To further determine whether NCAPG2 could promote PCa proliferation and invasion via c-MYC, we subsequently conducted colony formation, wound healing, and transwell assays. Our results showed that silencing NCAPG2 significantly reduced colony numbers, while c-MYC overexpression could compromise the inhibitory effect (Student’s t-test *P* < 0.05, Additional file 1: Figure S7a). Meanwhile, downregulation of NCAPG2 significantly impaired the migration ability of PCa cells, which was recovered by c-MYC upregulation (Student’s t-test *P* < 0.05, Additional file 1: Figure S7b). NCAPG2 downregulation also suppressed PCa cell invasion, while the effect can be reversed by restoring c-MYC expression (Student’s t-test *P* < 0.01, Additional file 1: Figure S7c). Similarly, NCAPG2 overexpression resulted in an enhancement of colony numbers, PCa cell migration and invasiveness, whereas c-MYC knockdown reversed above effects (Student’s t-test *P* < 0.05, Additional file 1: Figure S7d-f). Furthermore, IHC analysis of subcutaneous tumors indicated that NCAPG2 knockdown led to reduction in the level of c-MYC, following tumor shrinkage in vivo (Welch’s t-test *P* < 0.01, Additional file 1: Figure S7g).

### NCAPG2 activates c-MYC expression through a STAT3-mediated mechanism

**Fig. 7:**
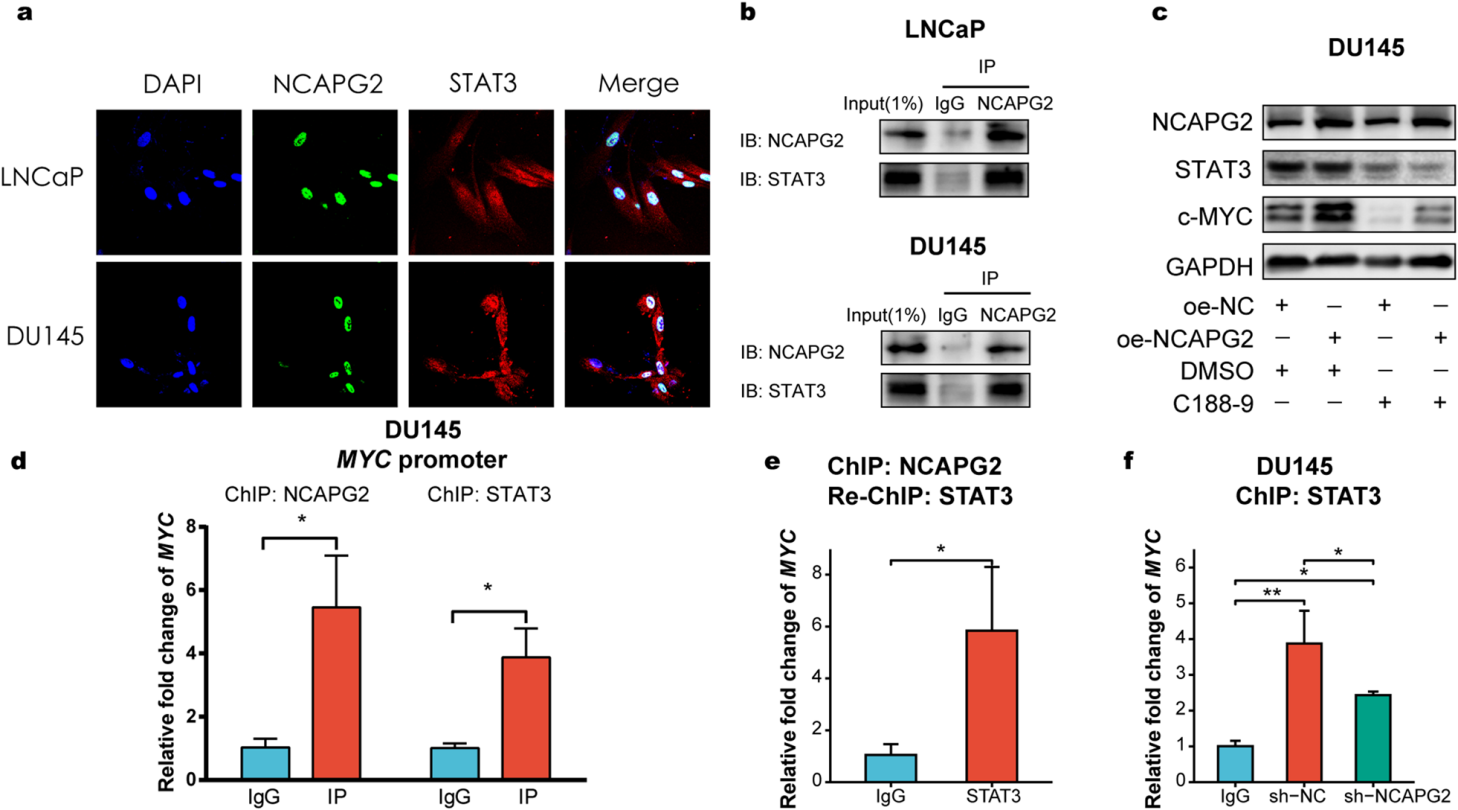
NCAPG2 activates c-MYC expression via STAT3-mediated mechanism. **a** Immunofluorescence showed the colocalization of NCAPG2 and STAT3 in the nucleus of PCa cells (LNCaP cells was pre-treated with IL-6 at a concentration of 20 ng/mL for 24 h). **b** Co-IP assays showed that NCAPG2 directly bound to STAT3. **c** WB assay revealed that upregulation of NCAPG2 enhanced the c-MYC expression, while treatment with C188-9 (STAT3 inhibitor) could effectively reverse this effect. **d** ChIP assays indicated that STAT3 and NCAPG2 occupied on the region of MYC promoter. **e** Re-ChIP analysis demonstrated NCAPG2 and STAT3 formed a complex and occupied on MYC promoter region. **f** ChIP assay showed that silencing NCAPG2 suppressed STAT3 binding to MYC promoter region. *PCa* prostate cancer, *co-IP* co-immunoprecipitation, *WB* western blot, *ChIP* chromatin immunoprecipitation

NCAPG2 was recently reported to directly bind with STAT3 to promote hepatocellular carcinoma [13]. Besides, it has been shown that STAT3 regulates the transcriptional activity of MYC gene in primitive acute myeloid leukemia cells [36], invoking the possibility that NCAPG2 might interact with STAT3 and subsequently activate c-MYC expression. Along these lines, IF assays were conducted and our results revealed the colocalization of NCAPG2 and STAT3 in the nucleus of PCa cells (Fig. 7a). Co-IP experiments demonstrated that there was a significant interaction between NCAPG2 and STAT3 (Fig. 7b). In addition, while overexpression of NCAPG2 increased the c-MYC level, this effect could be effectively reversed upon treatment with STAT3 inhibitor C188-9, as illustrated in Fig. 7c. ChIP analysis of DU145 cells further confirmed that NCAPG2 and STAT3 occupy and form a complex on the MYC promoter, as evidenced by re-ChIP analysis (Fig. 7d-e). Moreover, we observed that silencing NCAPG2 strongly attenuated STAT3 binding to MYC promoter (Fig. 7f), consistent with the involvement of NCAPG2 in promoting STAT3 transactivation. Therefore, our findings suggest that NCAPG2 binds to STAT3 and induces STAT3 occupancy of MYC promoter region, thereby activating c-MYC expression.

### NCAPG2 is related to PCa-CSC characteristics

**Fig. 8:**
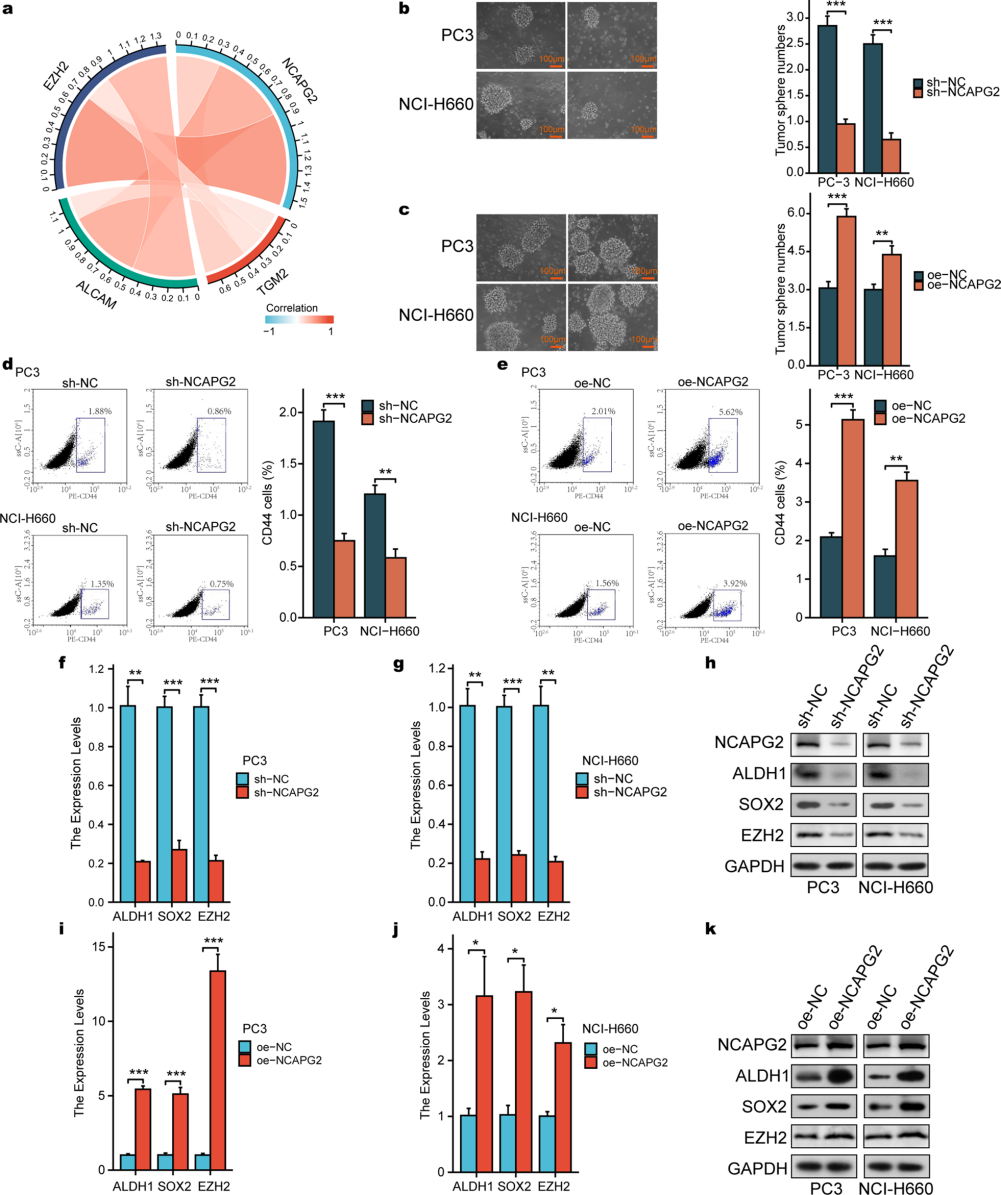
NCAPG2 is closely associated with PCa CSC characteristics. **a** Circos plot displaying the interconnectivity between NCAPG2 and stemness-related markers. **b**, **c** Representative images of sphere formation in PC3 and NCI-H660 cells and corresponding quantitative results. **d**, **e** Flow cytometry assay with CD44 in PCa cell lines and quantitative results. **f**-**k** The mRNA and protein expression of CSC makers (including ALDH1, SOX2, and EZH2) under the condition of NCAPG2 knockdown/upregulation in PC3 and NCI-H660 cells. *PCa* prostate cancer, *CSC* cancer stem cell. *P* values were calculated by the Student’s t-test. * means *P* < 0.05, ** means *P* < 0.01, *** means *P* < 0.001, and *P* < 0.05 is defined as statistically significant

**Fig. 9:**
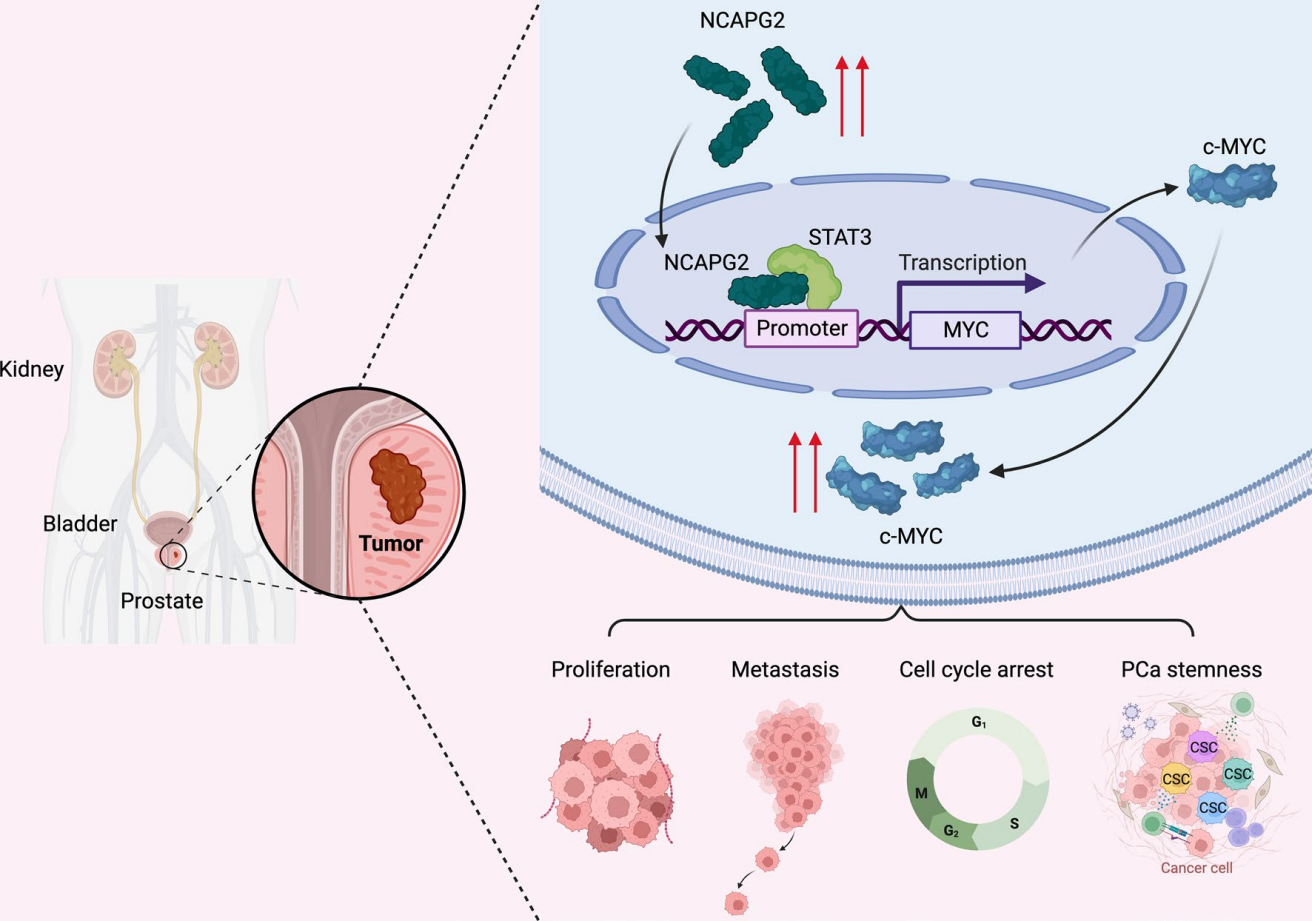
Schematic presentation of the mechanism underlying NCAPG2 regulation in PCa proliferation, metastasis and stemness. NCAPG2 directly bind to STAT3 and induce STAT3 occupancy on the MYC promoter, thus to transcriptionally activate c-MYC expression and finally promote PCa malignancy and drive cancer stemness

Given the regulatory relationship between NCAPG2 and c-MYC, and aberrant c-MYC overexpression has been linked to cancer cell stemness [37–39], we further explored the potential role of NCAPG2 in PCa-CSCs. Based on TCGA dataset, silico analysis was conducted and the results revealed a significant correlation between NCAPG2 and CSC markers, including *EZH2*, *ALCAM* and *TGM2* (Fig. 8a). To further verify whether NCAPG2 could confer PCa stemness, sphere-formation and flow cytometry assays were conducted in PC3 and NCI-H660 cells. As shown in Fig. 8b, c, the sphere-forming ability of both cell lines declined significantly following NCAPG2 knockdown, while NCAPG2 upregulation facilitated more tumor spheres. In addition, flow cytometry assay revealed that silencing NCAPG2 could decrease CD44+ subpopulations, whereas the aberrant overexpression of NCAPG2 led to a significant increase in the percentage of the CD44+ stem cell subpopulation (Fig. 8d-e). Furthermore, there was a positive correlation between NCAPG2 and several CSC markers (ALDH1, SOX2, and EZH2) in mRNA and protein levels (Student’s t-test *P* < 0.01, Fig. 8f-k). Finally, a schematic of the molecular mechanisms based on the above results is illustrated in Fig. 9.

## Discussion

NCAPG2 upregulation is associated with advanced pathological grade, lymph node metastasis, and poor prognosis in certain cancers [11–13]. However, the functional role of NCAPG2 and its clinical significance in PCa have not yet been investigated. In the present study, we demonstrated that NCAPG2 was upregulated at the mRNA and protein levels in PCa tissues based on public database and our patient cohort. Consistently, the expression of NCAPG2 was elevated in PCa cell lines compared with that in normal prostate stromal cell lines. Further, NCAPG2 levels were significantly associated with PCa progression and worse prognosis. Although the AUC values of NCAPG2 for a short-term (1 and 2-year) PFI survival ranged between 0.6 and 0.7 based on TCGA database, the survival analysis based on other databases (DKFZ2018, GSE70769, and MSKCC2010) showed a more significant prognostic value of NCAPG2 for PCa (Additional file 1: Figure S2g-i). The discrepancy may arise from (i) the variation in patient cohorts among different databases, (ii) the relatively limited efficacy of NCAPG2 as an early prognostic biomarker, which might be better combined with other parameters to enhance the predictive value.

A series of functional assays revealed that NCAPG2 could promote PCa cell proliferation, migration, and invasion, thus further strengthening the essential role for condensin complex components in tumor progression and cell cycle regulation [13, 40]. Moreover, our findings from the subcutaneous and orthotopic xenograft tumor models suggested that NCAPG2 knockdown was sufficient to significantly reduce tumor size. Since the underlying mechanism of NCAPG2 in PCa progression remains unknown, we transfected PCa cells with NCAPG2 overexpression plasmids and detected protein expression changes via TMT quantitative proteomics. Notably, c-MYC levels were significantly upregulated under NCAPG2 overexpression. To verify the relationship between NCAPG2 and c-MYC, we conducted GSEA for NCAPG2 in PCa patients from TCGA. The c-MYC activity was significantly and positively correlated with NCAPG2 expression. In LNCaP and DU145 cells, WB analysis results confirmed that NCAPG2 could upregulate the level of c-MYC.

To the best of our knowledge, no association has been reported previously between NCAPG2 and c-MYC expression in PCa. Herein, our functional assays demonstrated for the first time that NCAPG2 could promote PCa malignant biological properties via c-MYC. The c-MYC oncoprotein is a major regulator of gene transcription and an effective driver of carcinogenesis [41]. In normal cells, transcriptional regulation by c-MYC is highly controlled while being dysregulated in various cancers where it initiates and maintains tumor growth [42]. The amplification of MYC is one of the most common genomic alterations in PCa [43]. High c-MYC expression activates various downstream genes to drive PCa development and treatment resistance [44, 45].

We further sought to elucidate the mechanism by which NCAPG2 induces c-MYC expression. NCAPG2 was previously shown to directly interact with STAT3 and activate STAT3 signaling, thus driving hepatocellular carcinoma proliferation and metastasis [13]. As a latent transcription factor, STAT3 regulates a set of genes implicated in cancer cell survival, proliferation, angiogenesis, invasion, and metastasis [46]. Notably, Amaya et al. recently reported that STAT3 could occupy the MYC promoter region and enhance MYC to promote the survival of acute myeloid leukemia cells [36]. All above reports led us to hypothesize that NCAPG2 might form a complex with STAT3 on the c-MYC promoter to induce its expression. As a result, our IF and Co-IP assays verified the reciprocal interaction between NCAPG2 and STAT3 in PCa cells. More importantly, ChIP experiments demonstrated that NCAPG2 and STAT3 were recruited to the c-MYC promoter and cooperatively activated c-MYC expression, which was further supported by re-ChIP analysis. Therefore, our investigation provides a mechanistic basis for the contribution of NCAPG2 to PCa progression via the STAT3/c-MYC signaling pathway.

It has been well documented that PCa stemness contributes to lineage plasticity, poor response to androgen deprivation therapy, and the acquisition of aggressive phenotypes, such as neuroendocrine PCa [47, 48]. Moreover, emerging evidences indicate that MYC can act as a key regulator of cancer stem cells [23]. MYC is critical in controlling self-renewal, survival, and stem cell transformation in certain cancers [23, 38, 49]. Given the regulatory relationship between NCAPG2 and c-MYC, we investigated whether NCAPG2 is involved in driving PCa stemness. Our findings suggest that silencing NCAPG2 could not only inhibit the expression of CSC pluripotency markers, but also downregulate the self-renewal capacity of PCa cells. More recently, increasing emphasis has been placed on the development of direct c-MYC inhibitors for targeted therapy, with clinical trials in advanced cancers currently ongoing [50]. Nonetheless, owing to the intrinsically unstable conformations and essential physiological function of c-MYC, direct inhibition of c-MYC with small-molecule drugs still remains challenging [51, 52]. In this respect and of potential clinical relevance, targeting c-MYC key interaction partners, such as NCAPG2, and developing the specific small-molecular inhibitor may open new avenues for tackling PCa progression.

It is important to note that identifying the upstream regulators of NCAPG2 is also crucial for understanding the molecular mechanism of NCAPG2 in PCa. The origin of NCAPG2 upregulation and whether it could interact with multiple signal transduction pathways to promote PCa progression still remain obscure, which needs to be investigated in future studies.

## Conclusions

In conclusion, our study demonstrated that NCAPG2 is upregulated in PCa tissues, and its levels are significantly associated with PCa progression and poor prognosis. Moreover, NCAPG2 could promote PCa malignancy and drive cancer stemness through a previously unreported STAT3/c-MYC signaling-dependent mechanism, which provides a new direction for targeted PCa therapy.

### Supplementary Information

**Additional file 1: Figure S1.** Bioinformatics analysis of NCAPG2 expression in PCa based on TCGA and GTEx datasets. **a** Based on TCGA database, NCAPG2 showed a higher level in PCa tissues compared with normal tissues. **b** NCAPG2 expression was higher in PCa tissues than in paired paracancerous tissues from TCGA database. **c** Combined with TCGA and GTEx data, NCAPG2 exhibited a higher expression pattern in PCa tissues. **d**-**g** The expression of NCAPG2 in PCa patients with different tumor stages, different N stages, different grades of Gleason score and different levels of serum PSA. **h** NCAPG2 expression was higher in PCa patients with poor response after primary treatment. **i** NCAPG2 expression was higher in PCa patients with residual tumor after surgery. **j** PCa-specific death likelihood was greater for patients with higher expression of NCAPG2. **k** Patients with higher NCAPG2 expression in primary tumors had a significantly decreased PFI. *PCa* prostate cancer, *TCGA* The Cancer Genome Atlas, *GTEx* The Genotype-Tissue Expression, *CR* complete response, *PR* partial response, *SD* stable disease, *PD* progressive disease, *R0* no residual tumor, *R1* microscopic residual tumor, *R2* macroscopic residual tumor, *DSS* disease-specific survival, *PFI* progression-free interval, *ROC* receiver operating characteristic curve. *P* values were defined by the Wilcoxon test. * means *P* < 0.05, ** means *P* < 0.01, *** means *P* < 0.001, ns means *P* > 0.05, and *P* < 0.05 is defined as statistically significant. **Figure S2.** NCAPG2 had a good diagnostic and prognostic ability for PCa. **a**, **b** NCAPG2 yielded good ROC diagnostics in PCa from TCGA and GTEx databases. **c**-**f** The tdROC indicated that the level of NCAPG2 could effectively predict the 3-year, 6-year, 8-year and 10-year PFI survival of PCa patients, respectively. **g**-**i** PCa patients with high expression of NCAPG2 showed a poor BCR survival based on the DKFZ2018 database, GSE70769 database, and MSKCC2010 database. *PCa* prostate cancer, *TCGA* The Cancer Genome Atlas, *tdROC* time-dependent receiver operating characteristic curve, *PFI* progression-free interval, *BCR* biochemical recurrence. *P* < 0.05 is defined as statistically significant. **Figure S3.** Representative images of HE staining of PCa and para-carcinoma tissue sections. **Figure S4.** NCAPG2 overexpression promotes the proliferation, migration, invasion of PCa cells *in vitro*. **a**, **b** qPCR and WB analysis of NCAPG2 expression in LNCaP and DU145 cells after transfection of oe-NC or NCAPG2 plasmids. **c** In CCK-8 assay, both LNCaP and DU145 cells in oe-NCAPG2 group showed a faster proliferation status. **d** Colony formation assay showed that LNCaP and DU145 cells in oe-NCAPG2 group formed more colonies. **e**, **f** The migration rate and invasion ability of LNCaP and DU145 cells were enhanced with NCAPG2 overexpression, as shown by wound healing assay and transwell assay. *PCa* prostate cancer, *WB* western blot. *P* values were calculated by the Student’s t-test. * means *P* < 0.05, ** means *P* < 0.01, *** means *P* < 0.001, ns means *P* > 0.05, and *P* < 0.05 is defined as statistically significant. **Figure S5.** Representative images of HE staining for subcutaneous tumors (**a**) and WB analysis of NCAPG2 and p21 protein expression in the subcutaneous tumors (**b**-**e**). *HE* hematoxylin and eosin, *WB* western blot. *P* values were calculated by the Student’s t-test. *** means *P* < 0.001, and *P* < 0.05 is defined as statistically significant. **Figure S6.** NCAPG2 upregulation promotes the growth of PCa *in vivo*. **a** Representative images of subcutaneous xenograft derived from the oe-NCAPG2 and oe-NC PCa cells. **b**, **c** Tumor volume and body weight in nude mice of oe-NCAPG2 and oe-NC group were measured every 5 days. **d** The size and weights of subcutaneous tumors in the oe-NCAPG2 group were significantly larger than the oe-NC group. **e** Representative images of HE staining for subcutaneous xenograft derived from the oe-NC and oe-NCAPG2 PCa cells. **f**-**i** WB assays for NCAPG2 and p21 protein expression and quantitative results. *PCa* prostate cancer, *HE* hematoxylin and eosin, *WB* western blot. *P* values were calculated by the Student’s t-test. * means *P* < 0.05, ** means *P* < 0.01, *** means *P* < 0.001, and *P* < 0.05 is defined as statistically significant. **Figure S7.** NCAPG2 contributes to PCa malignancy through the mediation of c-MYC. **a** Knockdown of NCAPG2 inhibited the colonies formation of PCa cells (LNCaP and DU145), but overexpression of c-MYC could reverse the inhibitory effect. **b** Knockdown of NCAPG2 inhibited the migration ability of PCa cells, while overexpression of c-MYC could reverse above effect. **c** Knockdown of NCAPG2 inhibited invasiveness of PCa cells, while overexpression of c-MYC could reverse this inhibition. **d**-**f** NCAPG2 overexpression enhanced the colonies formation, PCa cell migration and invasiveness, while downregulation of c-MYC could reverse above effects. g. IHC results of subcutaneous tumor indicated that NCAPG2 knockdown led to reduction in the level of c-MYC. *PCa* prostate cancer, *IHC* immunohistochemical staining. *P* values were defined by Student’s t-test. * means *P* < 0.05, ** means *P* < 0.01, *** means *P* < 0.001, and *P* < 0.05 is defined as statistically significant.

**Additional file 2: Table S1.** The clinical information of 5 patients from Shengjing Hospital. **Table S2.** The clinical data of public databases used in this study. **Table S3.** The sequence of NCAPG2 shRNA and negative control Scramble. **Table S4.** The detailed information of primary antibodies in WB, IHC, IF, co-IP and ChIP experiments. **Table S5.** The primer sequences in qPCR procedure. **Table S6.** The primer sequences in ChIP qPCR procedure. **Table S7.** The baseline of PCa patients from TCGA in the study. **Table S8.** Identification of differentially expressed proteins in TMT quantitative proteomics.

## Data Availability

The datasets used and analyzed during the current study are available from the corresponding author upon reasonable request.

## Abbreviations

PCa: Prostate cancer
ROC: Receiver operating characteristic
co-IP: Co-immunoprecipitation
ChIP: Chromatin immunoprecipitation
TMT: Tandem mass tag
GSEA: Gene set enrichment analysis
PLK1: Polo-like kinase 1
CSC: Cancer stem cell
IF: Immunofluorescence
TCGA: The Cancer Genome Atlas
GTEx: The Genotype-Tissue Expression
TPM: Transcripts per million
tdROC: Time-dependent ROC
PFI: Progression-free interval
BCR: Biochemical recurrence
WB: Western blot
IHC: Immunohistochemistry
ROI: Region of interest
qPCR: Quantitative real-time polymerase chain reaction
CCK-8: Cell Counting Kit-8
KEGG: Kyoto Encyclopedia of Genes and Genomes
