# Supplementary figures and images for: NCAPG2 promotes prostate cancer malignancy and stemness via STAT3/c-MYC signaling
Source: J Transl Med. 2024 Jan 2;22:12. doi: 10.1186/s12967-023-04834-9 (PMC10763290; doi:10.1186/s12967-023-04834-9)

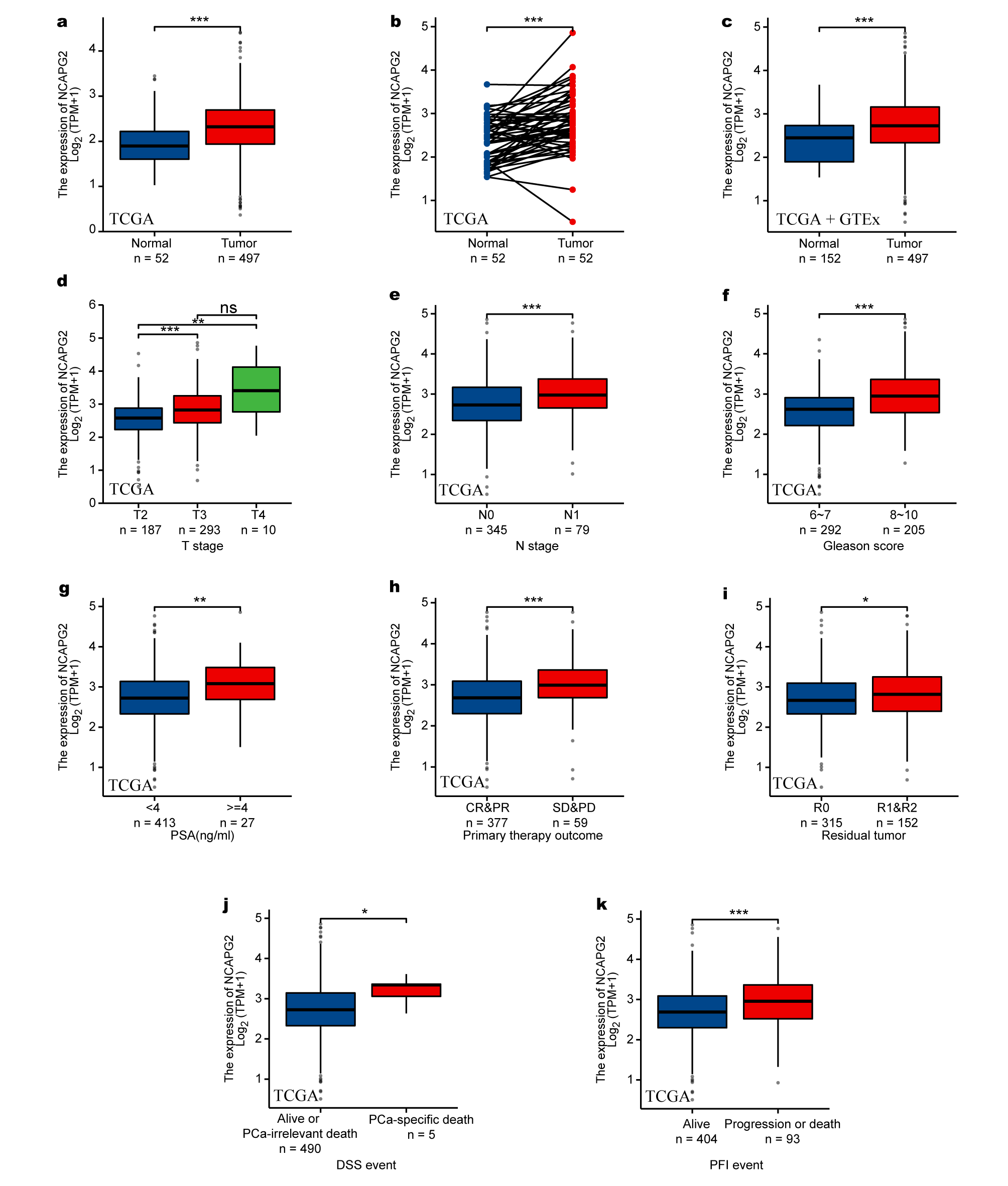

Supplement: Supplementary file 1 — Additional file 1: Figure S1. Bioinformatics analysis of NCAPG2 expression in PCa based on TCGA and GTEx datasets. a Based on TCGA database, NCAPG2 showed a higher level in PCa tissues compared with normal tissues. b NCAPG2 expression was higher in PCa tissues than in paired paracancerous tissues from TCGA database. c Combined with TCGA and GTEx data, NCAPG2 exhibited a higher expression pattern in PCa tissues. d-g The expression of NCAPG2 in PCa patients with different tumor stages, different N stages, different grades of Gleason score and different levels of serum PSA. h NCAPG2 expression was higher in PCa patients with poor response after primary treatment. i NCAPG2 expression was higher in PCa patients with residual tumor after surgery. j PCa-specific death likelihood was greater for patients with higher expression of NCAPG2. k Patients with higher NCAPG2 expression in primary tumors had a significantly decreased PFI. PCa prostate cancer, TCGA The Cancer Genome Atlas, GTEx The Genotype-Tissue Expression, CR complete response, PR partial response, SD stable disease, PD progressive disease, R0 no residual tumor, R1 microscopic residual tumor, R2 macroscopic residual tumor, DSS disease-specific survival, PFI progression-free interval, ROC receiver operating characteristic curve. P values were defined by the Wilcoxon test. * means P < 0.05, ** means P < 0.01, *** means P < 0.001, ns means P > 0.05, and P < 0.05 is defined as statistically significant. Figure S2. NCAPG2 had a good diagnostic and prognostic ability for PCa. a, b NCAPG2 yielded good ROC diagnostics in PCa from TCGA and GTEx databases. c-f The tdROC indicated that the level of NCAPG2 could effectively predict the 3-year, 6-year, 8-year and 10-year PFI survival of PCa patients, respectively. g-i PCa patients with high expression of NCAPG2 showed a poor BCR survival based on the DKFZ2018 database, GSE70769 database, and MSKCC2010 database. PCa prostate cancer, TCGA The Cancer Genome Atlas, tdROC time-depe [file 12967_2023_4834_MOESM1_ESM.zip › Additional file 1/Supplementary Figure 1.tif]

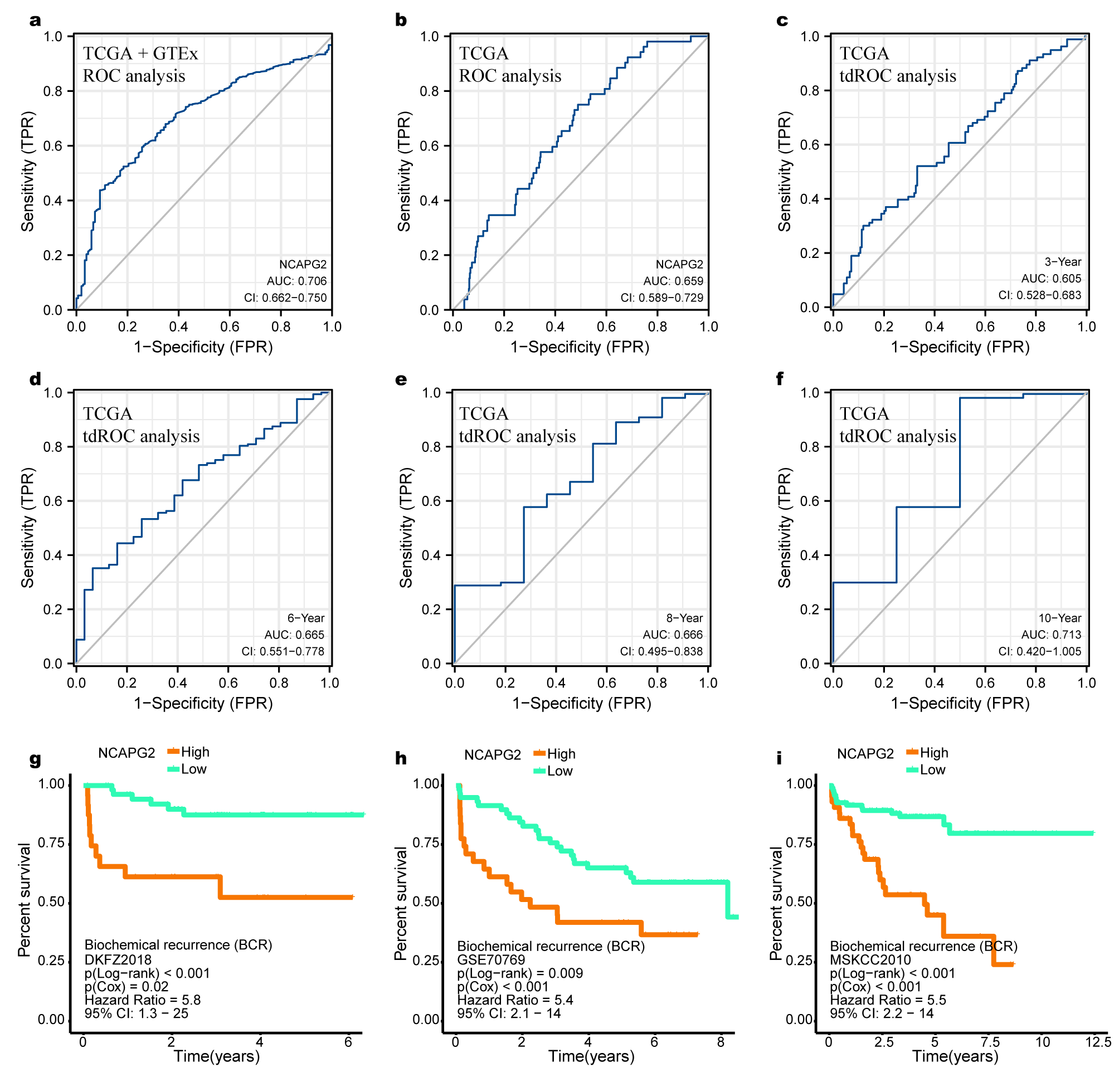

Supplement: Supplementary file 1 — Additional file 1: Figure S1. Bioinformatics analysis of NCAPG2 expression in PCa based on TCGA and GTEx datasets. a Based on TCGA database, NCAPG2 showed a higher level in PCa tissues compared with normal tissues. b NCAPG2 expression was higher in PCa tissues than in paired paracancerous tissues from TCGA database. c Combined with TCGA and GTEx data, NCAPG2 exhibited a higher expression pattern in PCa tissues. d-g The expression of NCAPG2 in PCa patients with different tumor stages, different N stages, different grades of Gleason score and different levels of serum PSA. h NCAPG2 expression was higher in PCa patients with poor response after primary treatment. i NCAPG2 expression was higher in PCa patients with residual tumor after surgery. j PCa-specific death likelihood was greater for patients with higher expression of NCAPG2. k Patients with higher NCAPG2 expression in primary tumors had a significantly decreased PFI. PCa prostate cancer, TCGA The Cancer Genome Atlas, GTEx The Genotype-Tissue Expression, CR complete response, PR partial response, SD stable disease, PD progressive disease, R0 no residual tumor, R1 microscopic residual tumor, R2 macroscopic residual tumor, DSS disease-specific survival, PFI progression-free interval, ROC receiver operating characteristic curve. P values were defined by the Wilcoxon test. * means P < 0.05, ** means P < 0.01, *** means P < 0.001, ns means P > 0.05, and P < 0.05 is defined as statistically significant. Figure S2. NCAPG2 had a good diagnostic and prognostic ability for PCa. a, b NCAPG2 yielded good ROC diagnostics in PCa from TCGA and GTEx databases. c-f The tdROC indicated that the level of NCAPG2 could effectively predict the 3-year, 6-year, 8-year and 10-year PFI survival of PCa patients, respectively. g-i PCa patients with high expression of NCAPG2 showed a poor BCR survival based on the DKFZ2018 database, GSE70769 database, and MSKCC2010 database. PCa prostate cancer, TCGA The Cancer Genome Atlas, tdROC time-depe [file 12967_2023_4834_MOESM1_ESM.zip › Additional file 1/Supplementary Figure 2.tif]

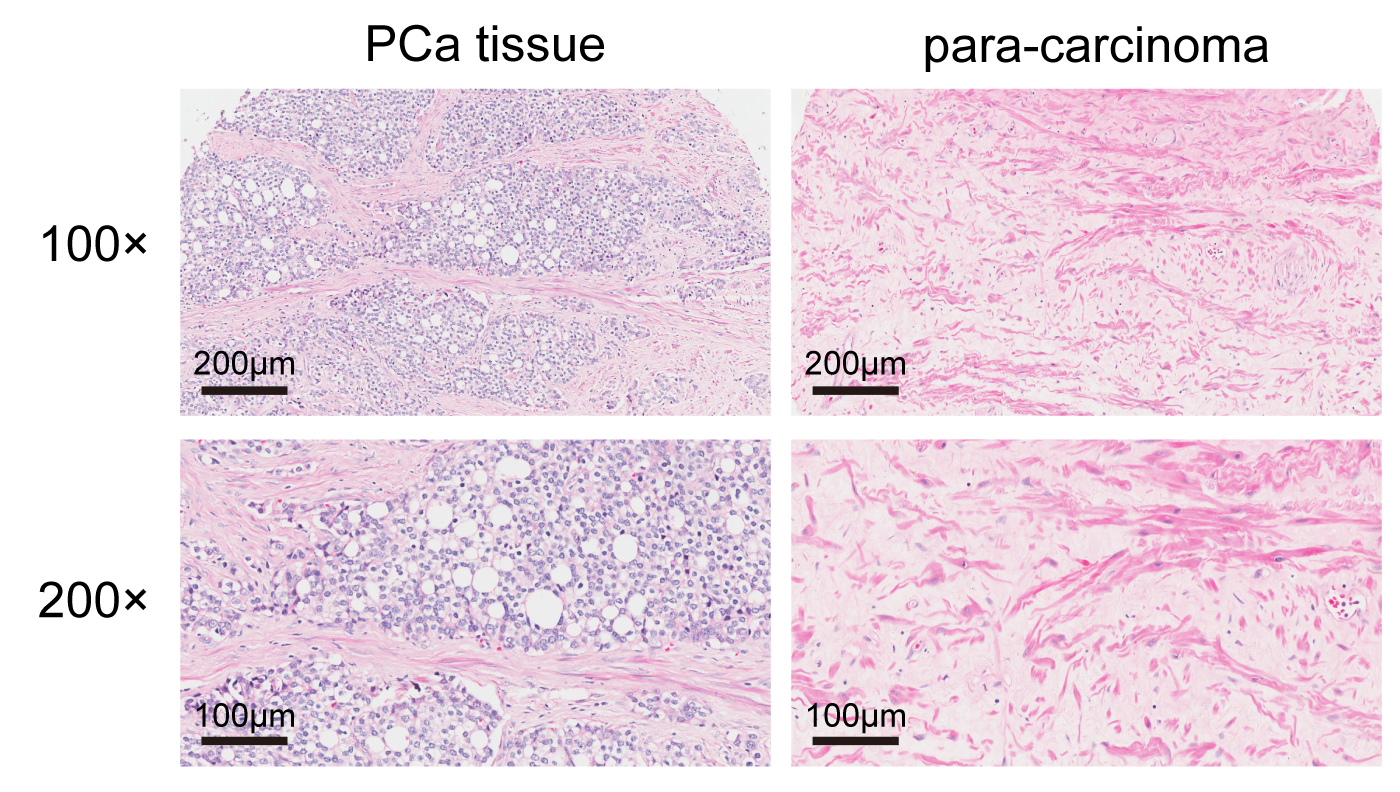

Supplement: Supplementary file 1 — Additional file 1: Figure S1. Bioinformatics analysis of NCAPG2 expression in PCa based on TCGA and GTEx datasets. a Based on TCGA database, NCAPG2 showed a higher level in PCa tissues compared with normal tissues. b NCAPG2 expression was higher in PCa tissues than in paired paracancerous tissues from TCGA database. c Combined with TCGA and GTEx data, NCAPG2 exhibited a higher expression pattern in PCa tissues. d-g The expression of NCAPG2 in PCa patients with different tumor stages, different N stages, different grades of Gleason score and different levels of serum PSA. h NCAPG2 expression was higher in PCa patients with poor response after primary treatment. i NCAPG2 expression was higher in PCa patients with residual tumor after surgery. j PCa-specific death likelihood was greater for patients with higher expression of NCAPG2. k Patients with higher NCAPG2 expression in primary tumors had a significantly decreased PFI. PCa prostate cancer, TCGA The Cancer Genome Atlas, GTEx The Genotype-Tissue Expression, CR complete response, PR partial response, SD stable disease, PD progressive disease, R0 no residual tumor, R1 microscopic residual tumor, R2 macroscopic residual tumor, DSS disease-specific survival, PFI progression-free interval, ROC receiver operating characteristic curve. P values were defined by the Wilcoxon test. * means P < 0.05, ** means P < 0.01, *** means P < 0.001, ns means P > 0.05, and P < 0.05 is defined as statistically significant. Figure S2. NCAPG2 had a good diagnostic and prognostic ability for PCa. a, b NCAPG2 yielded good ROC diagnostics in PCa from TCGA and GTEx databases. c-f The tdROC indicated that the level of NCAPG2 could effectively predict the 3-year, 6-year, 8-year and 10-year PFI survival of PCa patients, respectively. g-i PCa patients with high expression of NCAPG2 showed a poor BCR survival based on the DKFZ2018 database, GSE70769 database, and MSKCC2010 database. PCa prostate cancer, TCGA The Cancer Genome Atlas, tdROC time-depe [file 12967_2023_4834_MOESM1_ESM.zip › Additional file 1/Supplementary Figure 3.tif]

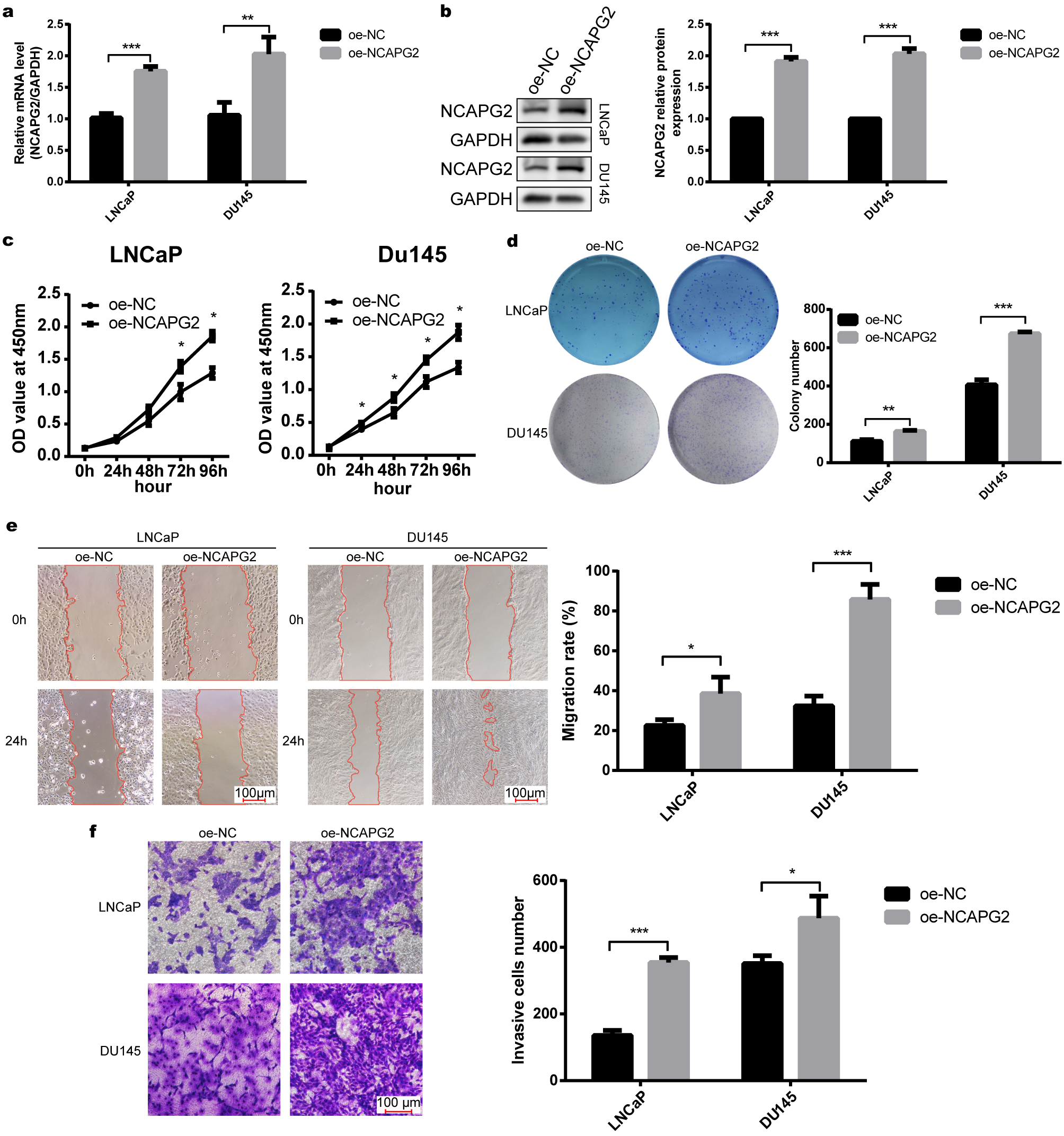

Supplement: Supplementary file 1 — Additional file 1: Figure S1. Bioinformatics analysis of NCAPG2 expression in PCa based on TCGA and GTEx datasets. a Based on TCGA database, NCAPG2 showed a higher level in PCa tissues compared with normal tissues. b NCAPG2 expression was higher in PCa tissues than in paired paracancerous tissues from TCGA database. c Combined with TCGA and GTEx data, NCAPG2 exhibited a higher expression pattern in PCa tissues. d-g The expression of NCAPG2 in PCa patients with different tumor stages, different N stages, different grades of Gleason score and different levels of serum PSA. h NCAPG2 expression was higher in PCa patients with poor response after primary treatment. i NCAPG2 expression was higher in PCa patients with residual tumor after surgery. j PCa-specific death likelihood was greater for patients with higher expression of NCAPG2. k Patients with higher NCAPG2 expression in primary tumors had a significantly decreased PFI. PCa prostate cancer, TCGA The Cancer Genome Atlas, GTEx The Genotype-Tissue Expression, CR complete response, PR partial response, SD stable disease, PD progressive disease, R0 no residual tumor, R1 microscopic residual tumor, R2 macroscopic residual tumor, DSS disease-specific survival, PFI progression-free interval, ROC receiver operating characteristic curve. P values were defined by the Wilcoxon test. * means P < 0.05, ** means P < 0.01, *** means P < 0.001, ns means P > 0.05, and P < 0.05 is defined as statistically significant. Figure S2. NCAPG2 had a good diagnostic and prognostic ability for PCa. a, b NCAPG2 yielded good ROC diagnostics in PCa from TCGA and GTEx databases. c-f The tdROC indicated that the level of NCAPG2 could effectively predict the 3-year, 6-year, 8-year and 10-year PFI survival of PCa patients, respectively. g-i PCa patients with high expression of NCAPG2 showed a poor BCR survival based on the DKFZ2018 database, GSE70769 database, and MSKCC2010 database. PCa prostate cancer, TCGA The Cancer Genome Atlas, tdROC time-depe [file 12967_2023_4834_MOESM1_ESM.zip › Additional file 1/Supplementary Figure 4.tif]

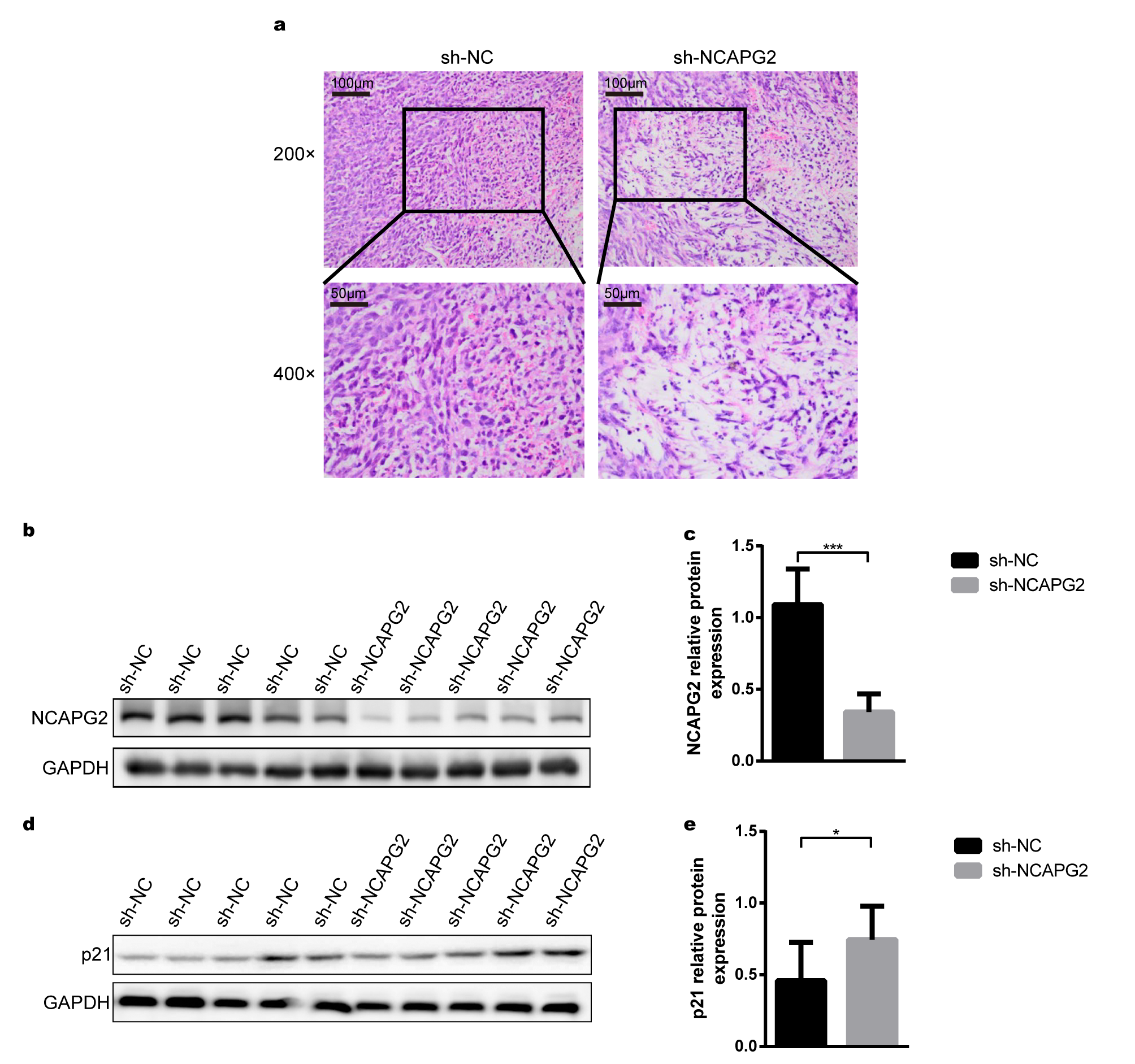

Supplement: Supplementary file 1 — Additional file 1: Figure S1. Bioinformatics analysis of NCAPG2 expression in PCa based on TCGA and GTEx datasets. a Based on TCGA database, NCAPG2 showed a higher level in PCa tissues compared with normal tissues. b NCAPG2 expression was higher in PCa tissues than in paired paracancerous tissues from TCGA database. c Combined with TCGA and GTEx data, NCAPG2 exhibited a higher expression pattern in PCa tissues. d-g The expression of NCAPG2 in PCa patients with different tumor stages, different N stages, different grades of Gleason score and different levels of serum PSA. h NCAPG2 expression was higher in PCa patients with poor response after primary treatment. i NCAPG2 expression was higher in PCa patients with residual tumor after surgery. j PCa-specific death likelihood was greater for patients with higher expression of NCAPG2. k Patients with higher NCAPG2 expression in primary tumors had a significantly decreased PFI. PCa prostate cancer, TCGA The Cancer Genome Atlas, GTEx The Genotype-Tissue Expression, CR complete response, PR partial response, SD stable disease, PD progressive disease, R0 no residual tumor, R1 microscopic residual tumor, R2 macroscopic residual tumor, DSS disease-specific survival, PFI progression-free interval, ROC receiver operating characteristic curve. P values were defined by the Wilcoxon test. * means P < 0.05, ** means P < 0.01, *** means P < 0.001, ns means P > 0.05, and P < 0.05 is defined as statistically significant. Figure S2. NCAPG2 had a good diagnostic and prognostic ability for PCa. a, b NCAPG2 yielded good ROC diagnostics in PCa from TCGA and GTEx databases. c-f The tdROC indicated that the level of NCAPG2 could effectively predict the 3-year, 6-year, 8-year and 10-year PFI survival of PCa patients, respectively. g-i PCa patients with high expression of NCAPG2 showed a poor BCR survival based on the DKFZ2018 database, GSE70769 database, and MSKCC2010 database. PCa prostate cancer, TCGA The Cancer Genome Atlas, tdROC time-depe [file 12967_2023_4834_MOESM1_ESM.zip › Additional file 1/Supplementary Figure 5.tif]

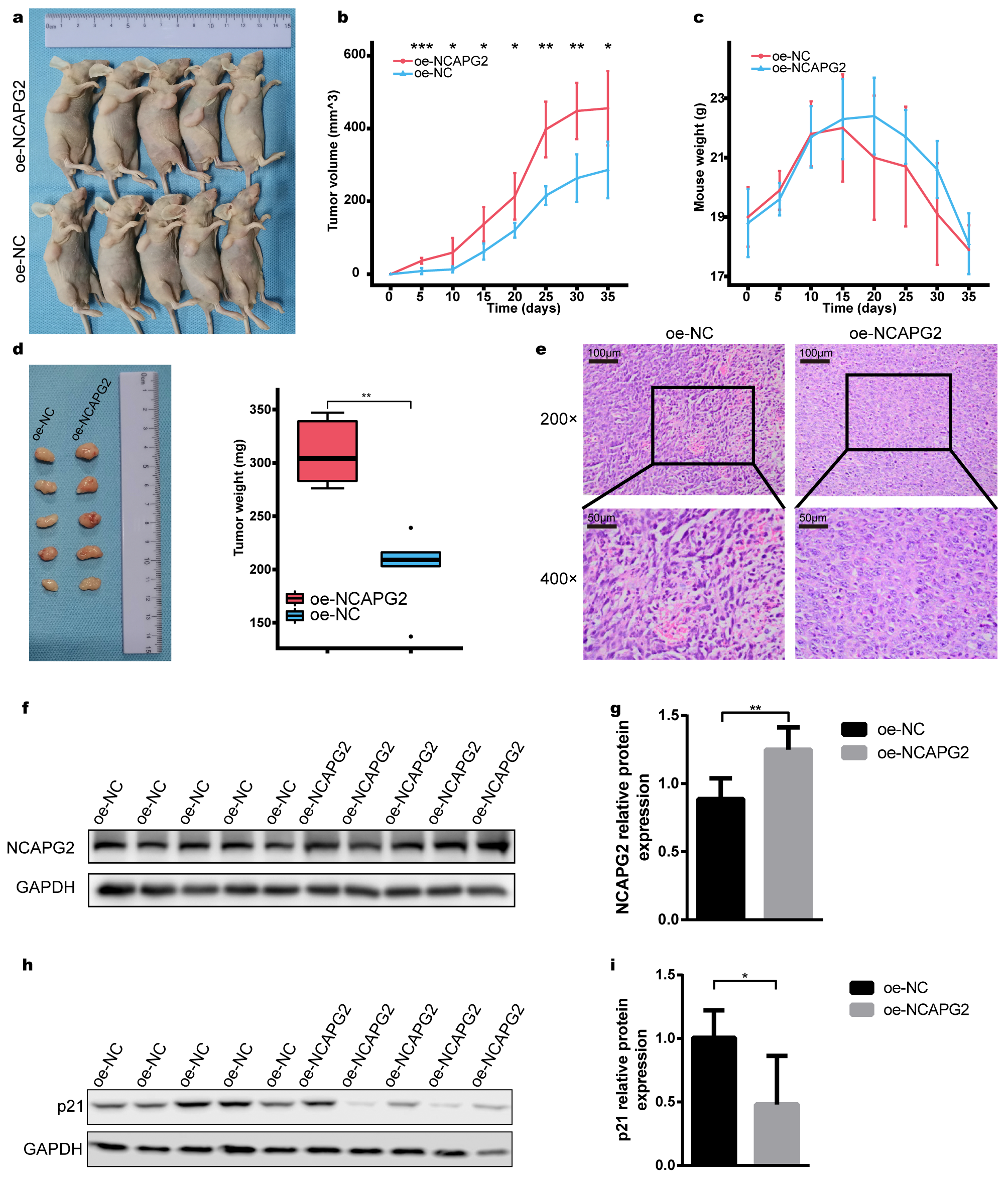

Supplement: Supplementary file 1 — Additional file 1: Figure S1. Bioinformatics analysis of NCAPG2 expression in PCa based on TCGA and GTEx datasets. a Based on TCGA database, NCAPG2 showed a higher level in PCa tissues compared with normal tissues. b NCAPG2 expression was higher in PCa tissues than in paired paracancerous tissues from TCGA database. c Combined with TCGA and GTEx data, NCAPG2 exhibited a higher expression pattern in PCa tissues. d-g The expression of NCAPG2 in PCa patients with different tumor stages, different N stages, different grades of Gleason score and different levels of serum PSA. h NCAPG2 expression was higher in PCa patients with poor response after primary treatment. i NCAPG2 expression was higher in PCa patients with residual tumor after surgery. j PCa-specific death likelihood was greater for patients with higher expression of NCAPG2. k Patients with higher NCAPG2 expression in primary tumors had a significantly decreased PFI. PCa prostate cancer, TCGA The Cancer Genome Atlas, GTEx The Genotype-Tissue Expression, CR complete response, PR partial response, SD stable disease, PD progressive disease, R0 no residual tumor, R1 microscopic residual tumor, R2 macroscopic residual tumor, DSS disease-specific survival, PFI progression-free interval, ROC receiver operating characteristic curve. P values were defined by the Wilcoxon test. * means P < 0.05, ** means P < 0.01, *** means P < 0.001, ns means P > 0.05, and P < 0.05 is defined as statistically significant. Figure S2. NCAPG2 had a good diagnostic and prognostic ability for PCa. a, b NCAPG2 yielded good ROC diagnostics in PCa from TCGA and GTEx databases. c-f The tdROC indicated that the level of NCAPG2 could effectively predict the 3-year, 6-year, 8-year and 10-year PFI survival of PCa patients, respectively. g-i PCa patients with high expression of NCAPG2 showed a poor BCR survival based on the DKFZ2018 database, GSE70769 database, and MSKCC2010 database. PCa prostate cancer, TCGA The Cancer Genome Atlas, tdROC time-depe [file 12967_2023_4834_MOESM1_ESM.zip › Additional file 1/Supplementary Figure 6.tif]

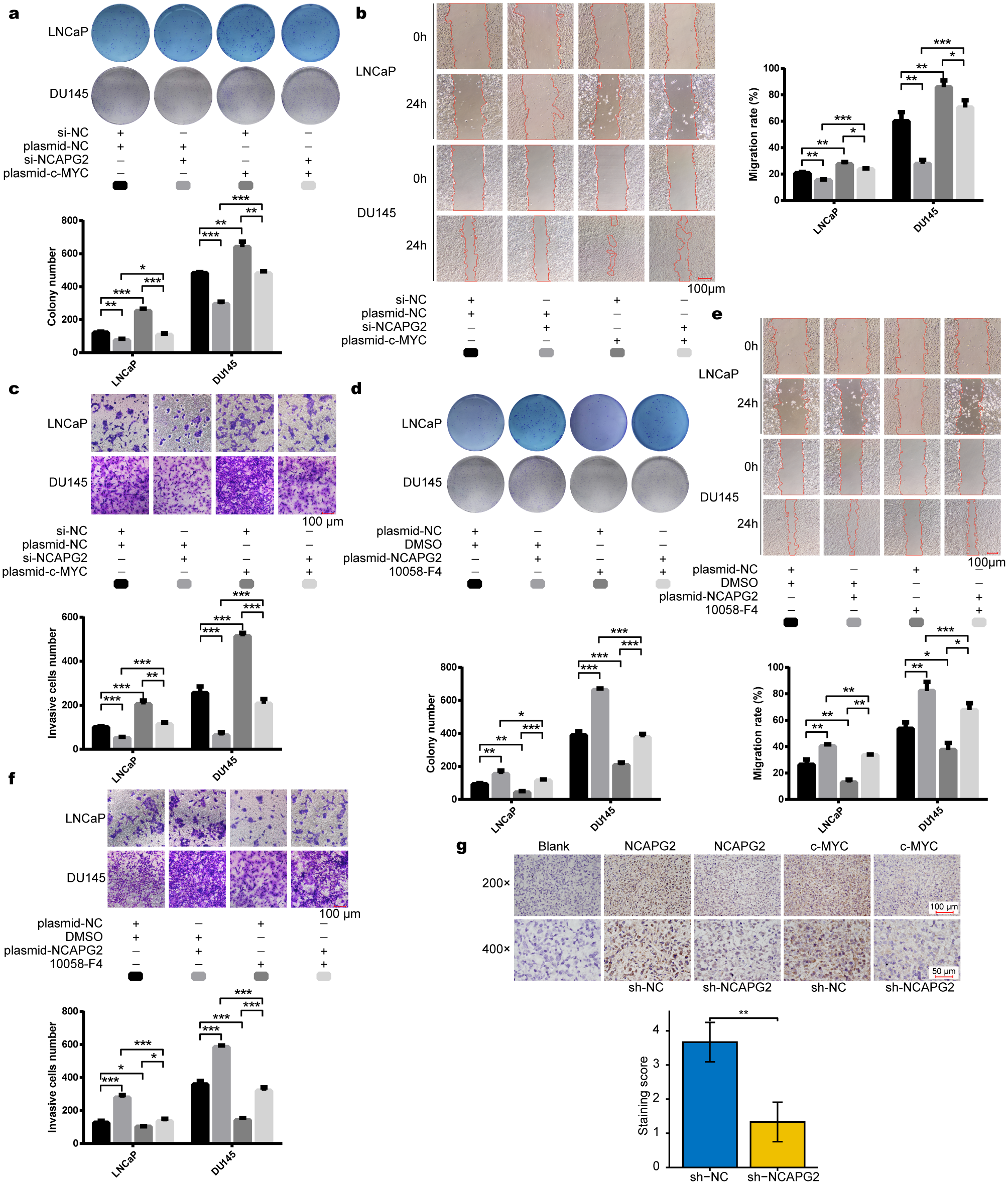

Supplement: Supplementary file 1 — Additional file 1: Figure S1. Bioinformatics analysis of NCAPG2 expression in PCa based on TCGA and GTEx datasets. a Based on TCGA database, NCAPG2 showed a higher level in PCa tissues compared with normal tissues. b NCAPG2 expression was higher in PCa tissues than in paired paracancerous tissues from TCGA database. c Combined with TCGA and GTEx data, NCAPG2 exhibited a higher expression pattern in PCa tissues. d-g The expression of NCAPG2 in PCa patients with different tumor stages, different N stages, different grades of Gleason score and different levels of serum PSA. h NCAPG2 expression was higher in PCa patients with poor response after primary treatment. i NCAPG2 expression was higher in PCa patients with residual tumor after surgery. j PCa-specific death likelihood was greater for patients with higher expression of NCAPG2. k Patients with higher NCAPG2 expression in primary tumors had a significantly decreased PFI. PCa prostate cancer, TCGA The Cancer Genome Atlas, GTEx The Genotype-Tissue Expression, CR complete response, PR partial response, SD stable disease, PD progressive disease, R0 no residual tumor, R1 microscopic residual tumor, R2 macroscopic residual tumor, DSS disease-specific survival, PFI progression-free interval, ROC receiver operating characteristic curve. P values were defined by the Wilcoxon test. * means P < 0.05, ** means P < 0.01, *** means P < 0.001, ns means P > 0.05, and P < 0.05 is defined as statistically significant. Figure S2. NCAPG2 had a good diagnostic and prognostic ability for PCa. a, b NCAPG2 yielded good ROC diagnostics in PCa from TCGA and GTEx databases. c-f The tdROC indicated that the level of NCAPG2 could effectively predict the 3-year, 6-year, 8-year and 10-year PFI survival of PCa patients, respectively. g-i PCa patients with high expression of NCAPG2 showed a poor BCR survival based on the DKFZ2018 database, GSE70769 database, and MSKCC2010 database. PCa prostate cancer, TCGA The Cancer Genome Atlas, tdROC time-depe [file 12967_2023_4834_MOESM1_ESM.zip › Additional file 1/Supplementary Figure 7.tif]
