# Supplementary material for: NCAPG2 promotes prostate cancer malignancy and stemness via STAT3/c-MYC signaling
Source: J Transl Med. 2024 Jan 2;22:12. doi: 10.1186/s12967-023-04834-9 (PMC10763290; doi:10.1186/s12967-023-04834-9)
Supplement: Supplementary file 2 — Additional file 2: Table S1. The clinical information of 5 patients from Shengjing Hospital. Table S2. The clinical data of public databases used in this study. Table S3. The sequence of NCAPG2 shRNA and negative control Scramble. Table S4. The detailed information of primary antibodies in WB, IHC, IF, co-IP and ChIP experiments. Table S5. The primer sequences in qPCR procedure. Table S6. The primer sequences in ChIP qPCR procedure. Table S7. The baseline of PCa patients from TCGA in the study. Table S8. Identification of differentially expressed proteins in TMT quantitative proteomics. [file 12967_2023_4834_MOESM2_ESM.zip › Additional file 2/Supplementary Table 3.docx]

Supplementary Table 3. The sequence of NCAPG2 shRNA and negative control Scramble.

| Target number | The internal serial number | Target sequence |
| --- | --- | --- |
| Human-NCAPG2-1 | Pbr17594 | CACGTTATTCGTCATTGCTTA |
| Human-NCAPG2-2 | Pbr17595 | AACGTCAAAGGCAGATCTGGA |
| Human-NCAPG2-3 | Pbr17596 | CTGCTCAGAAGGAAAGGTGTA |
| Scramble | Scramble | TTCTCCGAACGTGTCACGT |

The sequencing results of pbr17594-2:

CGGACGCTGTTAGAGAGAATTGGATTAATTTGACTGTAAACACAAAGATATTAGTACAAAATACGTGACGTAGAAAGTAA

TAATTTCTTGGGTAGTTTGCAGTTTTAAAATTATGTTTTAAAATGGACTATCATATGCTTACCGTAACTTGAAAGTATTT

CGATTTCTTGGCTTTATATATCTTGTGGAAAGGACGAAACACCGGCACGTTATTCGTCATTGCTTACTCGAGTAAGCAAT

GACGAATAACGTGTTTTTGAATTCGGATCCATTAGGCGGCCGCGTGGATAACCGTATTACCGCCATGCATTAGTTATTAA

TAGTAATCAATTACGGGGTCATTAGTTCATAGCCCATATATGGAGTTCCGCGTTACATAACTTACGGTAAATGGCCCGCC

TGGCTGACCGCCCAACGACCCCCGCCCATTGACGTCAATAATGACGTATGTTCCCATAGTAACGCCAATAGGGACTTTCC

ATTGACGTCAATGGGTGGAGTATTTACGGTAAACTGCCCACTTGGCAGTACATCAAGTGTATCATATGCCAAGTACGCCC

CCTATTGACGTCAATGACGGTAAATGGCCCGCCTGGCATTATGCCCAGTACATGACCTTATGGGACTTTCCTACTTGGCA

GTACATCTACGTATTAGTCATCGCTATTACCATGGTGATGCGGTTTTGGCAGTACATCAATGGGCGTGGATAGCGGTTTG

ACTCACGGGGATTTCCAAGTCTCCACCCCATTGACGTCAATGGGAGTTTGTTTTGGCACCAAAATCAACGGGACTTTCCA

AAATGTCGTAACAACTCCGCCCCATTGACGCAAATGGGCGGTAGGCGTGTACGGTGGGAGGTCTATATAAGCAGAGCTGG

TTTAGTGAACCGTCAGATCCGCTAGCGCTACCGGACGCCACCATGGTGAGCAAGGGCGAGGAGCTGTTCACCGGGGTGGT

GCCCATCCTGTCGAGCTGGACGGCGACGTAAACGGCACAAGTTCAGCGTGTCGGCGAGGGCGAGGCGATGCACTACGCAG

CTGACCTGAGTCATCTGCACACGCAGCTGCCCGTGCCCTACCACCCTCGTGACACCG

The sequencing results of pbr17595-1:

GCTAACGGCTGTTAGAGAGTAATTGGATTAATTTGACTGTAAACACAAAGATATTAGTACAAAATACGTGACGTAGAAAG

TAATAATTTCTTGGGTAGTTTGCAGTTTTAAAATTATGTTTTAAAATGGACTATCATATGCTTACCGTAACTTGAAAGTA

TTTCGATTTCTTGGCTTTATATATCTTGTGGAAAGGACGAAACACCGGAACGTCAAAGGCAGATCTGGACTCGAGTCCAG

ATCTGCCTTTGACGTTTTTTTGAATTCGGATCCATTAGGCGGCCGCGTGGATAACCGTATTACCGCCATGCATTAGTTAT

TAATAGTAATCAATTACGGGGTCATTAGTTCATAGCCCATATATGGAGTTCCGCGTTACATAACTTACGGTAAATGGCCC

GCCTGGCTGACCGCCCAACGACCCCCGCCCATTGACGTCAATAATGACGTATGTTCCCATAGTAACGCCAATAGGGACTT

TCCATTGACGTCAATGGGTGGAGTATTTACGGTAAACTGCCCACTTGGCAGTACATCAAGTGTATCATATGCCAAGTACG

CCCCCTATTGACGTCAATGACGGTAAATGGCCCGCCTGGCATTATGCCCAGTACATGACCTTATGGGACTTTCCTACTTG

GCAGTACATCTACGTATTAGTCATCGCTATTACCATGGTGATGCGGTTTTGGCAGTACATCAATGGGCGTGGATAGCGGT

TTGACTCACGGGGATTTCCAAGTCTCCACCCCATTGACGTCAATGGGAGTTTGTTTTGGCACCAAAATCAACGGGACTTT

CCAAAATGTCGTAACAACTCCGCCCCATTGACGCAAATGGGCGGTAGGCGTGTACGGTGGGAGGTCTATATAAGCAGAGC

TGGTTTAGTGAACCGTCAGATCCGCTAGCGCTACCGGACGCCACCATGGTGAGCAAGGGCGAGGAGCTGTTCACCGGGGT

GGTGCCCATCCTGGTCGAGCTGGACGGCGACGTAAACGGCACAAGTTCAGCGTGTCGGCGAGGGCGAGGGCGATGCCACC

TACGGCAGCTGACCCTGAGTTCATCTGCACAACGGCAGCTGCCCGTGCCCTGACCACCCTCG

The sequencing results of pbr17596-1:

ACTTATCCGGCTGTTAGAGAGTATTGGATTAATTTGACTGTAAACACAAAGATATTAGTACAAAATACGTGACGTAGAAA

GTAATAATTTCTTGGGTAGTTTGCAGTTTTAAAATTATGTTTTAAAATGGACTATCATATGCTTACCGTAACTTGAAAGT

ATTTCGATTTCTTGGCTTTATATATCTTGTGGAAAGGACGAAACACCGGCTGCTCAGAAGGAAAGGTGTACTCGAGTACA

CCTTTCCTTCTGAGCAGTTTTTGAATTCGGATCCATTAGGCGGCCGCGTGGATAACCGTATTACCGCCATGCATTAGTTA

TTAATAGTAATCAATTACGGGGTCATTAGTTCATAGCCCATATATGGAGTTCCGCGTTACATAACTTACGGTAAATGGCC

CGCCTGGCTGACCGCCCAACGACCCCCGCCCATTGACGTCAATAATGACGTATGTTCCCATAGTAACGCCAATAGGGACT

TTCCATTGACGTCAATGGGTGGAGTATTTACGGTAAACTGCCCACTTGGCAGTACATCAAGTGTATCATATGCCAAGTAC

GCCCCCTATTGACGTCAATGACGGTAAATGGCCCGCCTGGCATTATGCCCAGTACATGACCTTATGGGACTTTCCTACTT

GGCAGTACATCTACGTATTAGTCATCGCTATTACCATGGTGATGCGGTTTTGGCAGTACATCAATGGGCGTGGATAGCGG

TTTGACTCACGGGGATTTCCAAGTCTCCACCCCATTGACGTCAATGGGAGTTTGTTTTGGCACCAAAATCAACGGGACTT

TCCAAAATGTCGTAACAACTCCGCCCCATTGACGCAAATGGGCGGTAGGCGTGTACGGTGGGAGGTCTATATAAGCAGAG

CTGGTTTAGTGAACCGTCAGATCCGCTAGCGCTACCGGACGCCACCATGGTGAGCAAGGGCGAGGAGCTGTTCACCGGGG

TGGTGCCCATCCTGGTCGAGCTGGACGGCGACGTAACGGCCACAGTTCAGCGTGTCGGCGAGGGCGAGGGCGATGCCACC

TACGCAGCTGACCCTGAGTCATCTGCACACGGCAGCTGCCCGTGCCCTGACCACCCTCGTGACCACCCTGGACCTACGGC

GTGCAGTGCTT
