# Supplementary material for: NCAPG2 promotes prostate cancer malignancy and stemness via STAT3/c-MYC signaling
Source: J Transl Med. 2024 Jan 2;22:12. doi: 10.1186/s12967-023-04834-9 (PMC10763290; doi:10.1186/s12967-023-04834-9)
Supplement: Supplementary file 2 — Additional file 2: Table S1. The clinical information of 5 patients from Shengjing Hospital. Table S2. The clinical data of public databases used in this study. Table S3. The sequence of NCAPG2 shRNA and negative control Scramble. Table S4. The detailed information of primary antibodies in WB, IHC, IF, co-IP and ChIP experiments. Table S5. The primer sequences in qPCR procedure. Table S6. The primer sequences in ChIP qPCR procedure. Table S7. The baseline of PCa patients from TCGA in the study. Table S8. Identification of differentially expressed proteins in TMT quantitative proteomics. [file 12967_2023_4834_MOESM2_ESM.zip › Additional file 2/Supplementary Table 4.docx]

Supplementary Table 4. The detailed information of primary antibodies in WB, IHC, IF, co-IP and ChIP experiments.

| Gene symbol | Molecular weight | Application | Dilution | Production company | Catalog number |
| --- | --- | --- | --- | --- | --- |
| NCAPG2 | 131 kDa | WB | 1:2000 | Abcam | ab70350 |
| NCAPG2 | 131 kDa | IHC | 1:100 | Abcam | ab110882 |
| NCAPG2 | 131 kDa | IP | 6 μg/mg | Abcam | ab70350 |
| NCAPG2 | 131 kDa | IF | 1:50 | Sigma | HPA026631 |
| c-MYC | 49 kDa | WB | 1:5000 | Proteintech | 67447-1-Ig |
| c-MYC | 49 kDa | IHC | 1:500 | Proteintech | 67447-1-Ig |
| STAT3 | 88 kDa | IF | 1:300 | Proteintech | 60199-1-Ig |
| STAT3 | 88 kDa | IP | 4 μg/mg | Proteintech | 60199-1-Ig |
| STAT3 | 88 kDa | WB | 1:3000 | Proteintech | 60199-1-Ig |
| ALDH1 | 55 kDa | WB | 1:2000 | Proteintech | 15910-1-AP |
| SOX2 | 34 kDa | WB | 1:3000 | Proteintech | 66411-1-Ig |
| EZH2 | 86 kDa | WB | 1:5000 | Proteintech | 66476-1-Ig |
| p21 | 18kDa | WB | 1:200 | Invitrogen | 14-6715-81 |
| GAPDH | 37 kDa | WB | 1:3000 | Bioworld | AP0063 |
